# caBIG™ VISDA: Modeling, visualization, and discovery for cluster analysis of genomic data

**DOI:** 10.1186/1471-2105-9-383

**Published:** 2008-09-18

**Authors:** Yitan Zhu, Huai Li, David J Miller, Zuyi Wang, Jianhua Xuan, Robert Clarke, Eric P Hoffman, Yue Wang

**Affiliations:** 1 Department of Electrical and Computer Engineering, Virginia Polytechnic and State University, Arlington, VA 22203, USA; 2 Bioinformatics Unit, RRB, National Institute on Aging, NIH, Baltimore, MD 21224, USA; 3 Department of Electrical Engineering, The Pennsylvania State University, University Park, PA 16802, USA; 4 Research Center for Genetic Medicine, Children's National Medical Center, Washington, DC 20010, USA; 5 Department of Oncology, Physiology & Biophysics and Lombardi Comprehensive Cancer Center, Georgetown University, Washington, DC 20007, USA

## Abstract

**Background:**

The main limitations of most existing clustering methods used in genomic data analysis include heuristic or random algorithm initialization, the potential of finding poor local optima, the lack of cluster number detection, an inability to incorporate prior/expert knowledge, black-box and non-adaptive designs, in addition to the curse of dimensionality and the discernment of uninformative, uninteresting cluster structure associated with confounding variables.

**Results:**

In an effort to partially address these limitations, we develop the VIsual Statistical Data Analyzer (VISDA) for cluster modeling, visualization, and discovery in genomic data. VISDA performs progressive, coarse-to-fine (divisive) hierarchical clustering and visualization, supported by hierarchical mixture modeling, supervised/unsupervised informative gene selection, supervised/unsupervised data visualization, and user/prior knowledge guidance, to discover hidden clusters within complex, high-dimensional genomic data. The hierarchical visualization and clustering scheme of VISDA uses multiple local visualization subspaces (one at each node of the hierarchy) and consequent subspace data modeling to reveal both global and local cluster structures in a "divide and conquer" scenario. Multiple projection methods, each sensitive to a distinct type of clustering tendency, are used for data visualization, which increases the likelihood that cluster structures of interest are revealed. Initialization of the full dimensional model is based on first learning models with user/prior knowledge guidance on data projected into the low-dimensional visualization spaces. Model order selection for the high dimensional data is accomplished by Bayesian theoretic criteria and user justification applied via the hierarchy of low-dimensional visualization subspaces. Based on its complementary building blocks and flexible functionality, VISDA is generally applicable for gene clustering, sample clustering, and phenotype clustering (wherein phenotype labels for samples are known), albeit with minor algorithm modifications customized to each of these tasks.

**Conclusion:**

VISDA achieved robust and superior clustering accuracy, compared with several benchmark clustering schemes. The model order selection scheme in VISDA was shown to be effective for high dimensional genomic data clustering. On muscular dystrophy data and muscle regeneration data, VISDA identified biologically relevant co-expressed gene clusters. VISDA also captured the pathological relationships among different phenotypes revealed at the molecular level, through phenotype clustering on muscular dystrophy data and multi-category cancer data.

## Background

Due to limited existing biological knowledge at the molecular level, clustering has become a popular and effective method to extract information from genomic data. Genomic data clustering may help to discover novel functional gene groups, gene regulation networks, phenotypes/sub-phenotypes, and developmental/morphological relationships among phenotypes [1-7]. Due to the complex and challenging nature of the task, various clustering algorithms have been applied in genomic data analysis [5,8-10], including statistical, model-based methods [11-13], "nonparametric" graph-theoretic approaches [14,15], stability analysis based consensus clustering [16], agglomerative/divisive hierarchical clustering [2], and partitional methods, such as Self-Organizing Maps (SOM) [1,17] and K-Means Clustering (KMC) [18]. The assignment of data points to clusters can also be either hard (exclusive) or soft (partial), the latter achieved by fuzzy clustering [19,20] and mixture modeling [11-13].

While there is a rich variety of existing methods, unfortunately when clustering genomic data, most of them suffer from several major limitations, which we summarize as follows. (1) Clustering methods such as KMC and mixture model fitting are sensitive to the quality of model initialization and may converge to poor local optima of the objective function, which will yield inaccurate clustering outcomes, especially when applied to genomic datasets that have high dimensionality and small sample size [21-25]. (2) Stability/reproducibility of clustering outcomes is also a critical issue [5,23,26-28]. Some clustering methods, such as HC, may not give reproducible clustering outcomes in the presence of small dataset perturbations, additive noise, or outliers [8,22]. (3) For statistical, model-based approaches, traditional information-theoretic model selection criteria, such as Minimum Description Length (MDL) [29,30], may grossly fail in estimating the cluster number due to inaccurate parameter estimation resulting from the "curse of dimensionality" or due to too many freely adjustable parameters [21,31]. As one alternative solution, stability analysis has been applied for model selection [32-34]. (4) Unsupervised informative gene selection for sample clustering is a critical, difficult problem due to the existence of many irrelevant genes respective to the phenotypes/sub-phenotypes of interest [9,10,35]. Existing iterative algorithms wrapping gene selection around sample clustering were developed and tested for the two-cluster case [13,36]. More research effort targeting multi-cluster unsupervised gene selection is needed. (5) Confounding variables produce clustering structure that may not be associated with the biological processes of interest. Effective removal or compensation for confounding influences still requires further research efforts [5,35]. (6) Most clustering algorithms do not utilize prior knowledge, although some semi-supervised clustering methods do exploit gene annotations to help construct gene clusters [12,37,38]. Besides database knowledge, the user's domain knowledge and human intelligence assisted by data visualization can also help to produce accurate and meaningful clustering outcomes for practical tasks [39,40]. For example, hierarchical data visualization schemes based on mixture models with human-data interaction were developed [41-43]. (7) Many clustering algorithms have a non-adaptive nature, without a mechanism for incorporating and taking advantage of results from other methods or user knowledge. These algorithms may fail badly without a "backup plan" when the algorithm's underlying statistical or geometric cluster assumptions are violated. (For the benefit of readers, we expand on each of these limitations in section 1 of Additional file 1.)

To address some of the existing methods' limitations outlined above and design a comprehensive and flexible clustering tool effectively applicable to cluster modeling, visualization, and discovery on genomic data, we developed a hierarchical data exploration and clustering approach, the VIsual Statistical Data Analyzer (VISDA). VISDA performs progressive, divisive hierarchical clustering and visualization, supported by hierarchical mixture modeling, supervised/unsupervised informative gene selection, supervised/unsupervised data projection, and user/prior knowledge guidance, to discover hidden clusters within complex, high-dimensional genomic data. The data exploration process in VISDA starts from the top level where the whole dataset is viewed as a cluster, with clusters then hierarchically subdivided in successive layers until all salient structure in the data is revealed. Since a single 2-D data projection, even if it is nonlinear, may be insufficient for revealing all cluster structures in multimodal, high dimensional data, the hierarchical visualization and clustering scheme of VISDA uses multiple local projection subspaces (one at each node of the hierarchy) and consequent subspace data modeling to reveal both global and local cluster structures. Consistent with the "divide and conquer" principle, each local data projection and modeling can be fulfilled with relatively simple method/model, while the complete hierarchy maintains overall flexibility and conveys considerable clustering information.

The inclusive VISDA framework readily incorporates the advantages of various complementary data clustering and visualization algorithms to visualize the obtained clusters, which not only give a "transparent" clustering process that can enhance the user's understanding of the data structure, but also provide an interface to incorporate human intelligence (e.g. user's discernment of sub-cluster separability and outliers) and domain knowledge to help improve clustering accuracy and avoid finding nonmeaningful or confounding cluster structure. Specifically, the interactive user participation guides the coarse-to-fine cluster discover via (1) the selection of a local visualization, from a suite of data projections, each sensitive to a distinct type of data structure, for best revealing a cluster's substructure; (2) user-directed parameter initialization for the new sub-clusters that divide existing clusters; (3) user-guided model order selection, applied in conjunction with MDL, for deciding the number of sub-clusters in the local visualization space.

Based on its complementary building blocks and flexible functionality, VISDA is comprehensively suitable for multiple genomic data clustering tasks, including gene clustering, sample clustering, and phenotype clustering (wherein phenotype labels for samples are known), albeit with customized modifications for each of these tasks. Specifically, VISDA's sample clustering requires dimensionality reduction via unsupervised informative gene selection, whereas the phenotype clustering algorithm exploits the knowledge of phenotype labels in performing supervised informative gene selection, supervised data visualization, and statistical modeling that preserves the unity of samples from the same phenotype, which fulfils that in phenotype clustering known phenotypes, i.e. groups of samples with the same phenotype label, are taken as data objects to be clustered. An important goal of phenotype clustering is to discover a Tree Of Phenotypes (TOP), i.e. a hierarchical tree structure with all phenotypes as leaves of the tree, which may reflect important biological relationships among the phenotypes.

In this paper, we show that VISDA gives stable and improved clustering accuracy compared to several benchmark clustering methods, i.e. conventional agglomerative Hierarchical Clustering (HC) [2], KMC [18], SOM [1,17], and Standard Finite Normal Mixtures (SFNM) fitting [11,22]. Its model order selection scheme is also shown to be effective on high dimensional data clustering. VISDA detects critical co-expressed gene clusters associated with specific genomic functions or gene regulation networks in muscular dystrophy and muscle regeneration studies. It also captures the pathological relationships between phenotypes reflected at the mRNA level, through phenotype clustering on muscular dystrophy data and multi-category cancer data. VISDA is a toolkit of caBIG™ [44] and has been successfully applied on several biomedical research projects [3,6,7,45,46]. The open source caBIG™ VISDA software package is free available at . Matlab code implementing a full functional version is free available at .

## Methods

In this section, we first introduce the main steps of VISDA algorithm that directly describe the complete VISDA processing for the task of gene clustering. Next, we extend the algorithm to work on sample clustering by adding unsupervised informative gene selection as a data pre-processing step. Finally, we extend the algorithm for phenotype clustering by incorporating a cluster visualization and decomposition scheme that explicitly utilizes the phenotype category information.

### VISDA algorithm

**Figure 1:**
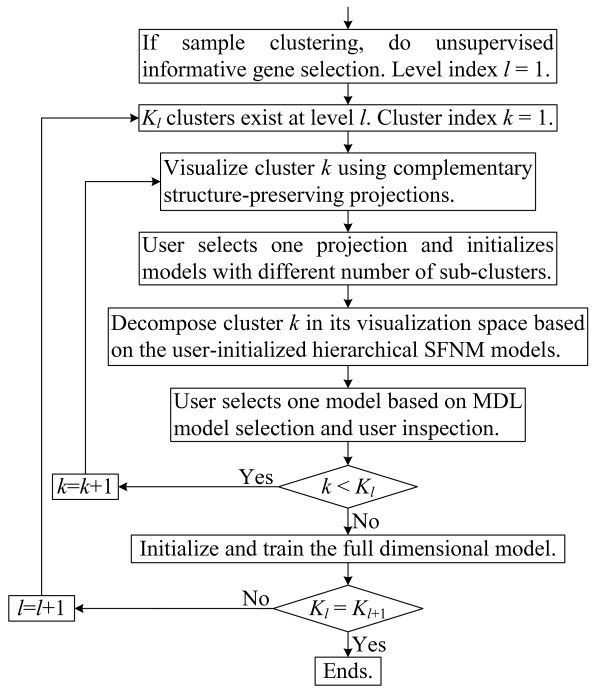
VISDA's flowchart.

Let **t **= {**t**_1_, **t**_2_,..., **t**_*N*_|**t**_*i *_∈ R^*p*^, *i *= 1, 2,..., *N*} denote *N p*-dimensional data points to be clustered. Based on a hierarchical SFNM model, VISDA performs top-down divisive clustering as outlined in Figure 1. Major blocks in the flowchart are introduced in the following subsections. Suppose that the hierarchical exploration has already proceeded to the *l*th level, i.e. *K*_*l *_clusters have already been detected at level *l *and the posterior probability of data point **x***i *belonging to cluster *k *(*k *= 1,..., *K*_*l*_) is *z*_*i*, *k*_.

#### Visualization of cluster *k *by complementary structure-preserving projections

For cluster *k*, VISDA projects the given cluster onto 2-D spaces by five projection methods preserving different data structures associated with distinct types of sub-cluster tendency.

(1) Principal Component Analysis (PCA) [22]. Sub-cluster structure is consistent with variation within the cluster. PCA does an eigenvalue decomposition of the cluster's covariance matrix. The PCA projection uses the eigenvectors associated with the largest two eigenvalues as the projection directions. Measured by second order statistics, the PCA projection preserves the largest variation within the cluster.

(2) Principal Component Analysis – Projection Pursuit Method (PCA-PPM) [45]. Although sub-cluster structure will surely impart variation within the cluster, the directions of largest variation will not always best reveal the sub-cluster structure [47]. Projection pursuit calculates the kurtosis of the projected data distribution on each of the eigenvectors obtained by PCA. If kurtosis is large, the projected data distribution presents a single sharp peak, which indicates no sub-cluster structure [47]. PCA-PPM projection selects the two eigenvectors whose associated kurtoses are smallest as projection directions.

(3) Locality Preserving Projection (LPP) [48]. In LPP, the projection directions are obtained by minimizing a compactness cost function, which is a weighted summation of the pair-wise square distances between points in the projection space. The square distances between neighboring points are given large weights while the square distances between far apart points are given small weights. Thus the minimization emphasizes keeping the neighboring data points close in the projection space to preserve the local data structure. The minimization is achieved by the generalized eigenvalue decomposition [48]. The eigenvectors are orthogonalized by the Gram-Schmidt process [49] to form an affine projection matrix.

(4) HC-KMC-SFNM-DCA [7]. DCA refers to Discriminatory Component Analysis, a supervised mode projection (dimension reduction) method aiming to preserve as much as possible the discrimination/separability between known classes [45]. The main idea behind HC-KMC-SFNM-DCA is to use an unsupervised clustering method to obtain a partition of the data and then use DCA as a visual validation of partition separability. If a partition of the data is indeed consistent with the sub-cluster structure, the consequent DCA projection will show distinct sub-clusters. The first three steps in HC-KMC-SFNM-DCA sequentially execute HC, KMC and SFNM fitting, and use the clustering result of the previous step to initialize the next one. When performing HC, the user chooses a distance threshold to cut the cluster into sub-clusters on the dendrogram, which initializes sub-clusters and determines the sub-cluster number. After obtaining the partition, we take the obtained sub-clusters as known classes and use DCA to present the separability among them. DCA here maximizes the weight Fisher criterion [50], which is a modified version of the Fisher criterion that is the trace of the multiplication of the inversed within-class scatter matrix and the between-class scatter matrix in the projection space [22]. Compared to the Fisher criterion, the weighted Fisher criterion weights the class pairs in the between-class scatter matrix, thus confines the influence of class pairs that are well-separated, and emphasizes the class pairs that are overlapped to improve over-all separation of the classes [50]. The maximization of the weighted Fisher criterion is achieved through eigenvalue decomposition and the two eigenvectors with the largest eigenvalues are orthogonalized by the Gram-Schmidt process [49] to form an affine projection matrix. Please see section 2.1.1 of Additional file 1 for formulas of maximizing the weighted Fisher criterion.

(5) Affinity Propagation Clustering – Discriminatory Component Analysis (APC-DCA) [22,51]. Similar to HC-KMC-SFNM-DCA, APC-DCA follows the idea of using DCA to evaluate/confirm partitions learned in an unsupervised manner, but based on a different clustering procedure. By viewing each data point as a node in a network, APC recursively transmits along edges of the network real-valued messages, whose magnitudes reflect the current affinity that one data point has for choosing another data point as its sub-cluster center, until a good set of sub-cluster centers and corresponding sub-clusters emerges [51]. The messages are updated to search for minima of the cost function, which is the sum of dissimilarities between data points and their sub-cluster centers. It was shown that the affinity propagation method finds the best solution amongst those in a particularly large proximal region in the solution space [51,52].

In each of the five projection methods, after the projection matrix **W**_*k *_for cluster *k *is determined, the data projection is achieved by

(1) $$x_{i}=W_{k}^{T}\left(t_{i}-\mu_{t,k}\right),$$

where **x**_*i *_is the image of data point *i *in the projection space, and **μ**_**t**, *k *_is the mean of cluster *k *in the original data space. The subscript '**t**' indicates that these parameters model the data in the high-dimensional original data space. Each point is displayed with an intensity proportional to the posterior probability *z*_*i*, *k *_(or we can set a threshold, and only points with posterior probabilities bigger than this threshold are displayed). Available prior/domain information about the data is also provided to the user via additional user interface. For gene clustering, prior information can be gene annotations, such as gene ID and the functional category. For sample clustering, prior information can be array annotations, such as the experimental condition under which the array was generated.

Each of these five projection methods preserves different yet complementary data structure associated with a distinct type of sub-cluster tendency. PCA preserves directions with largest variation in the data. PCA-PPM moderates PCA to consider projection directions on which the projected data have flat distributions or distributions with thick tails. LPP preserves the neighborhood structure of the data. HC-KMC-SFNM-DCA and APC-DCA directly target presenting discrimination among sub-clusters via different unsupervised partition approaches. HC-KMC-SFNM partitioning is model-based and allows the user to determine the sub-cluster number, while APC partitioning is nonparametric and automatically determines the sub-cluster number. Because each projection method has its own, distinct theoretical or experimental assumption of data structure associated with sub-clusters, while whether the underlying sub-clusters of interest are consistent with these assumptions is data/application dependent, using all of them simultaneously increases the likelihood that sub-clusters of interest are revealed.

After inspecting all five projections, the user is asked to select one projection that best reveals the sub-cluster structure as the final visualization. Human interaction in choosing the best projection (and hence substructure) provides an interface to incorporate human discernment and domain knowledge in cluster visualization, which gives potential to avoid confounding, irrelevant, and uninteresting substructures. The selection of a suitable/good projection is data/application dependent. Several guidelines based on human discernment and prior knowledge are as follows: (1) Select a projection in which the sub-clusters are well-separated and show clear sub-cluster structure. (2) Select a projection in which no sub-clusters are simply composed of several outliers. (3) Select a projection that does not oppose prior knowledge, i.e. if the user is certain about the relationship between some genes/samples under the particular experimental condition that produced the data, he/she can choose a projection that favours this relationship. In addition, when the data size is pretty large, PCA and PCA-PPM may be preferred over HC-KMC-SFNM-DCA, LPP, and APC-DCA, because the latter three projection algorithms have much higher computational complexity. More details, discussion, and empirical understanding of these projections can be found in section 2.1.1 of Additional file 1.

#### Decomposition of cluster *k *in its visualization space based on the hierarchical SFNM model

We use the two-level hierarchical SFNM model to present the relationship between the *l*th and the *l *+ 1th levels of VISDA's hierarchical exploration. The probability density function for a two-level hierarchical SFNM model is formulated as:

(2) $$\begin{array}{l} \text{f}\left(t_{i}|\theta_{t},\pi \right)=\sum_{k=1}^{K_{l}}\pi_{k}\sum_{j=1}^{K_{k,l+1}}\pi_{j|k}\text{ g}\left(t_{i}|\theta_{t,(k,j)}\right)\\ \begin{array}{cc} \sum_{k=1}^{K_{l}}\pi_{k}=1 & \sum_{j=1}^{K_{k,l+1}}\pi_{j|k}=1 \end{array} \end{array},$$

where *K*_*k*, *l*+1 _sub-clusters exist at level *l *+ 1 for each cluster *k *at level *l*, *π*_*k *_is the mixing proportion for cluster *k *at level *l*, *π*_*j*|*k *_is the mixing proportion for sub-cluster *j *within cluster *k*, g(•) is the Gaussian probability density function, and **θ**_**t**, (*k*, *j*) _are the associated parameters of sub-cluster *j*. We focus on the decomposition of cluster *k *in its visualization space. To achieve this, we maximize the expectation of the conditional log-likelihood of the sub-cluster model, i.e.

(3) $$\text{L}(x|\theta_{x,k},\pi_{k},z_{k})=\sum_{i=1}^{N}z_{i,k}\ln\left(\sum_{j=1}^{K_{k,l+1}}\pi_{j|k}\text{ g}\left(x_{i}|\theta_{x,\left(k,j\right)}\right)\right),$$

where **x **= {**x**_1_, **x**_2_,..., **x**_*N*_|**x**_*i *_∈ R^2^, *i *= 1, 2,..., *N*} are the projected points in the visualization space, subscript '**x**' indicates these parameters model data in the visualization space, and **θ**_**x**, (*k*, *j*) _are the associated parameters of sub-cluster *j*. Equation (3) is a weighted log-likelihood, which can be maximized or locally maximized by the Expectation Maximization (EM) algorithm [53]. The EM algorithm performs the E-step and M-step iteratively until convergence. The E-step and M-step for training the hierarchical SFNM model are given by

(4) $$\begin{array}{ll} \text{E Step}: & z_{i,\left(k,j\right)}=z_{i,k}\frac{\pi_{j|k}\text{ g}(x_{i}|\mu_{x,\left(k,j\right)},\Sigma_{x,\left(k,j\right)})}{\sum_{j=1}^{K_{k,l+1}}\pi_{j|k}\text{ g}(x_{i}|\mu_{x,\left(k,j\right)},\Sigma_{x,\left(k,j\right)})}\\ \text{M Step}: & \begin{array}{l} \begin{array}{cc} \pi_{j|k}=\sum_{i=1}^{N}z_{i,\left(k,j\right)}/\sum_{i=1}^{N}z_{i,k}, & \mu_{x,\left(k,j\right)}=\sum_{i=1}^{N}z_{i,\left(k,j\right)}x_{i}/\sum_{i=1}^{N}z_{i,\left(k,j\right)} \end{array}\\ \Sigma_{x,\left(k,j\right)}=\sum_{i=1}^{N}z_{i,\left(k,j\right)}\left(x_{i}-\mu_{x,\left(k,j\right)}\right){\left(x_{i}-\mu_{x,\left(k,j\right)}\right)}^{T}/\sum_{i=1}^{N}z_{i,\left(k,j\right)} \end{array} \end{array},$$

where *z*_*i*, (*k*, *j*) _is the posterior probability of data point **x**_*i *_belonging to the *j*th sub-cluster in cluster *k*, **μ**_**x**, (*k*, *j*) _and **Σ**_**x**, (*k*, *j*) _are the mean and covariance matrix of sub-cluster *j *in cluster *k*. This training process decomposes cluster *k *by keeping the data point's posterior probability of belonging to cluster *k *unchanged and adjusting its conditional posterior probabilities of belonging to the lower level sub-clusters. For details and derivations of the model and the algorithm, please see section 2.1.2 of Additional file 1.

To get an accurate and biologically meaningful initialization of the model parameters, which is a key factor for obtaining a good clustering result, VISDA utilizes human initialization of sub-cluster means in the visualization space. The user pinpoints on the visualization screen where he/she thinks the sub-cluster means should be, according to his/her discernment of the sub-cluster structure and domain knowledge. This initialization gives the potential to avoid learning uninteresting or biologically irrelevant substructures. For example, if a sub-cluster has several outliers, the user will most likely initialize the sub-cluster mean on the "main body" of the sub-cluster but not on the outliers.

Models with different numbers of sub-clusters are initialized by the user and trained by the EM algorithm. The obtained partitions of all the models are displayed to the user as a reference for model selection. The MDL criterion is also utilized as a theoretical validation for model selection [29,30]. When the data size is small, the classical MDL model selection with Gaussian distributions has the tendency to select complex models in low dimensional space [54]. Based on our experimental experience and reference to [54,55], we use a modified formula to calculate the description length given by

(5) $$\begin{array}{l} -\text{L}(x|\theta_{x,k},\pi_{k},z_{k})+\frac{K_{a}\times N_{k}\times \ln\left(N_{k}\right)}{2\left(N_{k}-K_{a}\right)}\\ \begin{array}{cc} N_{k}=\sum_{i=1}^{N}z_{i,k} & K_{a}=6K_{k,l+1}-1 \end{array} \end{array},$$

where *K*_*a *_and *N*_*k *_are the number of freely adjustable parameters and the effective number of data points in the cluster, respectively, and L(**x**|**θ**_***x***, *k*_, *π*_*k*_, **z**_*k*_) is given in Equation (3). This modified MDL formula not only eases the trend to overestimate the sub-cluster number when the data size is small, but also is asymptotically consistent with the classical MDL formula introduced in section 2.1.2 of Additional file 1. To fully take advantage of human intelligence and domain knowledge, the user is allowed to override the MDL model selection by specifying a sub-cluster number based on his/her inspection of all the partitions resulting from models with different number of sub-clusters. Again, this offers an opportunity to incorporate human intelligence and domain knowledge in the clustering process. For example, in gene clustering, the user can choose a model in which genes known to be co-regulated and co-expressed in the particular experiment fall in the same sub-cluster, even although the model's description length may not be the smallest, or the user can refuse a model with sub-clusters formed by a few outliers.

#### Initialization and training of the full dimensional model

Each sub-cluster in the chosen model corresponds to a new cluster at the *l *+ 1th level of the hierarchical exploration. The parameters of the sub-clusters in all the selected models of *l*th level are transformed from the visualization spaces back to the original data space. This transform is achieved by

(6) $$\begin{array}{l} \mu_{t,\left(k,j\right)}=W_{k}\mu_{x,\left(k,j\right)}+\mu_{t,k}\\ \Sigma_{t,\left(k,j\right)}=W_{k}\Sigma_{x,\left(k,j\right)}W_{k}^{T} \end{array},$$

where **μ**_**t**, (*k*, *j*) _and **Σ**_**t**, (*k*, *j*) _are the mean and covariance matrix for the *j*th sub-cluster of cluster *k *in the original data space, and **W**_*k *_is the projection matrix for cluster *k*, as introduced in Equation (1). Obviously, these transformed parameters may not accurately describe the full dimensional distribution. From Equation (2), we can see that the two-level hierarchical SFNM model can be written in the form of a standard SFNM model simply by putting *π*_*k *_inside the second summation, giving a mixture with components indexed by (*k*, *j*) and mass *π*_*k*_*π*_*j*|*k*_. Thus we can use the transformed parameters as initialization for the SFNM model in the original data space and then further train this model using the EM algorithm to refine the parameters. Formulas for the SFNM model and the corresponding EM algorithm are given in section 2.1.3 of Additional file 1. When this training finishes, the *l *+ 1th level in the exploration hierarchy is generated. If no new clusters are detected at level *l*+1 compared to level *l*, or if the user believes all interesting cluster structure has been detected, the algorithm ends.

### Algorithm extension for sample clustering

The main clustering and visualization algorithm introduced above is directly applicable for gene clustering, which is a "data-sufficient" case due to the large ratio of gene number to sample number. Sample clustering is usually a "data-insufficient" case that suffers from the "curse of dimensionality", because in sample clustering the number of data objects to be clustered is much smaller than the data dimensionality. Many of the genes are actually irrelevant respective to the phenotypes/sub-phenotypes of interest [9,10,35]. Thus we perform unsupervised informative gene selection as a preprocessing step before we use the above algorithm to cluster the samples. Non-informative genes can be divided into two categories. (1) Irrelevant genes, i.e. those which do not respond to the physiological event. These genes are normally constantly expressed over the experimental conditions. (2) Non-discriminative genes, i.e. ones that do not contribute to cluster structure.

Two variation criteria, the variance and the absolute difference between the minimum and maximum gene expression values across all the samples, can be used to identify and then remove constantly expressed genes. For each criterion, a rank of all the genes is obtained, with genes of large variation ranked at the top.

Discrimination power analysis measures each gene's individual ability both to elicit and to discriminate clusters/components. These components are generated by fitting a 1-D SFNM model to the gene's expression values using the EM algorithm. To determine the component number, we followed the iterative procedure in [56]. The 1-D SFNM model is initialized with a high model order (much bigger than the true component number), with randomly chosen means and uniform variances. In each iteration, we (one-by-one) trial-delete each component and rerun the fitting algorithm. The component whose removal yields minimum description length will be permanently removed. This iterative process ends when only one component remains, and the component number is determined by the MDL principle via comparing the description lengths of solutions in the sequence. Once the SFNM models are trained, genes with a single component are removed, because they do not support any cluster structure. For the other genes, the accuracy in classifying samples to components resulting from applying the Maximum A Posteriori probability (MAP) rule quantifies the gene's discrimination power. The MAP rule assigns a sample to the component that it most likely belongs to, evaluated by the posterior probability learned through the SFNM fitting. Thus, a rank of genes according to their discrimination power can be constructed.

Based on the variation ranks and the discrimination power rank, a list of genes with large variations and large discrimination power can be obtained by taking the intersection of the top parts of the ranks. Further details about these three gene ranking schemes can be found in section 2.2 of Additional file 1.

### Algorithm extension for phenotype clustering

**Figure 2:**
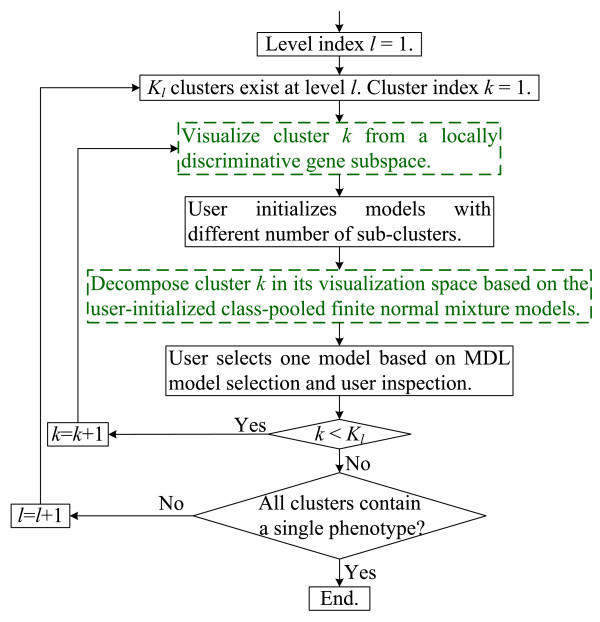
**The flowchart including the algorithm extension for phenotype clustering**. The green blocks with dashed borders indicate the algorithm extensions, i.e. the modified visualization scheme and decomposition scheme.

As an extension of the main clustering and visualization algorithm, phenotype clustering follows a similar hierarchical, interactive exploration process, shown in Figure 2. Since the phenotype categories are known, cluster visualization and decomposition can exploit this information, which leads to the modified visualization and decomposition scheme indicated by the green blocks with dashed borders in Figure 2. Suppose that the exploration process has proceeded to the *l*th level with *K*_*l *_phenotype clusters, each of which contains all the samples from one or multiple phenotypes. For phenotype cluster *k *(*k *= 1,..., *K*_*l*_), if it contains only one phenotype, we do not need to decompose it; if it contains two phenotypes, we simply split the cluster into two sub-clusters, each containing the samples of one phenotype; if it contains more than two phenotypes, we do the following to visualize and decompose it. Let *Q*_*k *_and *N*^(*q*) ^denote the number of phenotypes in cluster *k *and the number of samples from the *q*th phenotype in cluster *k*, respectively.

#### Visualization of cluster *k *from a locally discriminative gene subspace

We first use supervised discriminative gene selection to form a locally discriminative gene subspace respective to the phenotype categories in the cluster. The locally discriminative gene subspace contains the most discriminative genes, where the discrimination power of a gene is measured by

(7) $$\begin{array}{ccc} \left(\sum_{q=1}^{Q_{k}-1}\sum_{m=q+1}^{Q_{k}}r_{q}r_{m}{\left(\mu_{q}-\mu_{m}\right)}^{2}\right)/\left(\sum_{q=1}^{Q_{k}}r_{q}\sigma_{q}^{2}\right), & \text{and} & r_{q}=N^{\left(q\right)}/\sum_{q=1}^{Q_{k}}N^{\left(q\right)} \end{array},$$

where *r*_*q *_is the sample proportion of phenotype *q *in cluster *k*, *μ*_*q *_and *σ *_*q *_are the mean and standard deviation of the gene's expression values in phenotype *q*. The number of genes in this gene subspace is *Q*_*k*_*n*_*g*_, where *n*_*g *_is the number of selected genes per phenotype, an input parameter of the algorithm. We use Discriminatory Component Analysis (DCA) to project the samples from the gene subspace onto a 2-D visualization space. Because an important outcome of phenotype clustering is the relative relationships among the phenotypes that are estimated directly based on the relative distances between samples of different phenotypes, to preserve the original and undistorted data structure, DCA here maximizes the Fisher criterion that treats all the phenotype pairs equally. The Fisher criterion is calculated based on the known phenotype categories. Maximization of the Fisher criterion is achieved by eigenvalue decomposition and the projection matrix is obtained by orthogonalizing the eigenvectors associated with the largest two eigenvalues [22,49]. When the samples are projected onto the visualization space, prior information in the form of phenotype labels of samples are also provided to the user. For further details and formulas, please see section 2.3.1 of Additional file 1.

#### Decomposition of cluster *k *in its visualization space based on the class-pooled finite normal mixture model

Phenotype clustering differs from sample/gene clustering in that it assigns a cluster label to each phenotype in its entirety (all samples therefrom), not to each sample/gene. Based on this difference, we use a class-pooled finite normal mixture to model the projected samples in the visualization space. Let {**x**^(1)^,..., **x**^(*q*)^,..., $x^{\left(Q_{k}\right)}$} denote the projected phenotypes, where **x**^(*q*) ^= {$x_{i}^{\left(q\right)}$, *i *= 1, 2,..., *N*^(*q*)^} is the set of samples from phenotype *q*. The probability density function for all samples from phenotype *q *is

(8) $$\begin{array}{ccc} \text{f}\left(x^{\left(q\right)}|\theta_{x},\pi \right)=\sum_{j=1}^{K_{k,l+1}}\pi_{j}\prod_{i=1}^{N^{\left(q\right)}}\text{g}\left(x_{i}^{\left(q\right)}|\theta_{x,j}\right) & \text{and} & \sum_{j=1}^{K_{k,l+1}}\pi_{j}=1 \end{array},$$

where cluster *k *at level *l *is decomposed into *K*_*k*, *l*+1 _sub-clusters at level *l *+ 1, *π*_*j *_and **θ**_**x**, *j *_are the mixing proportion and parameters associated with sub-cluster *j*. The model parameters are learned by the EM algorithm using the formulas introduced in section 2.3.2 of Additional file 1.

Similar to sample/gene clustering, the user is asked to initialize the sub-cluster means by pinpointing them on the visualization screen according to his/her understanding about the data structure and domain knowledge. Models with different numbers of sub-clusters are initialized by the user, and trained by the EM algorithm. The resulting partitions are shown to the user for comparison. The MDL model selection criterion is also applied for theoretical validation. Details and formulas of MDL model selection can be found in section 2.3.2 of Additional file 1. The user can override the MDL model selection by specifying the number of sub-clusters according to his/her justification and domain knowledge. Once the best model is selected, the phenotypes are assigned to sub-clusters using the MAP rule.

After visualizing and decomposing the clusters at level *l*, all the sub-clusters become clusters at level *l *+ 1. Thus the hierarchical exploration process proceeds to the *l *+ 1th level. If all the clusters at the *l *+ 1th level contain a single phenotype, the algorithm ends.

### A demo application of VISDA on sample clustering

**Figure 3:**
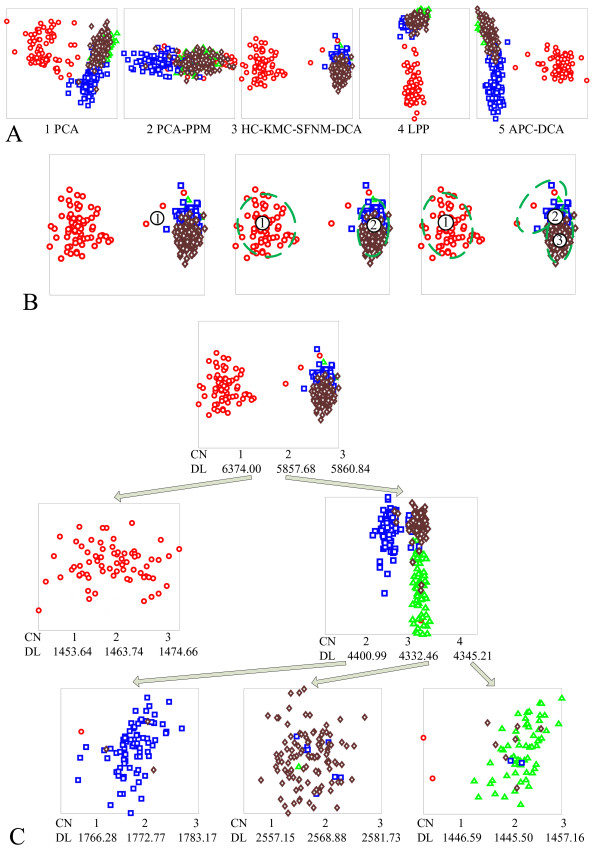
**An illustration of VISDA on sample clustering**. (a) The five different projections obtained at the top level. Red circles are brain cancer; green triangles are colon cancer; blue squares are lung cancer; and brown diamonds are ovary cancer. (b) The user's initialization of cluster means (indicated by the numbers in the small circles) and the resulted clusters (indicated by the green dashed ellipses). The left, middle, and right figures are for the models of one cluster, two clusters, and three clusters, respectively. (c) The hierarchical data structure detected by VISDA. Sub-Cluster Number (CN) and corresponding Description Length (DL) are shown under the visualization.

To show how VISDA discovers data structure, we consider the UM microarray gene expression cancer dataset as an example and perform sample clustering [57]. This dataset consists of brain (73 samples), colon (60 samples), lung (91 samples), ovary (119 samples, 113 for ovarian cancer and 6 for uterine cancer), and pancreas (10 samples) cancer classes. We removed the pancreas category due to its relatively small size. The total number of genes is 7069. We applied our unsupervised gene selection method to choose informative genes. To emphasize the genes with discrimination power, those which manifest true, underlying cluster structure, we used a more stringent requirement on a gene's discrimination power than on its variation. We took the intersection of the top 700 discriminative genes, the top 1600 genes ranked by variance, and the top 1600 genes from the absolute difference ranking. A set of 107 genes was thus obtained and used as the input gene space for sample clustering. Clustering of the 343 samples was performed in a purely unsupervised fashion, i.e. category labels and the number of categories were not used by the algorithm and were not known to the user during the clustering process. After clustering, we use colours to indicate different cancer categories, with the results shown in Figure 3.

Figure 3a shows the five different projections obtained at the top level. PCA gives roughly a three-cluster structure with dispersion of the left cluster and some overlap between the other two clusters. PCA-PPM shows a two-cluster structure with significant overlap. The HC-KMC-SFNM-DCA projection gives a well-separated two-cluster structure. LPP also produces a two-cluster structure, but not well-separated. APC-DCA gives roughly a three-cluster structure with dispersion of the right cluster and overlap between the other two clusters. For the sake of simplicity, we select data visualization based on human inspection of the separability among the clusters. Since the HC-KMC-SFNM-DCA projection presents the best separated clusters, we select it for the top level visualization and continue decomposing these clusters. Figure 3b shows the user's initialization and corresponding obtained partitions of models with different cluster number for the top level decomposition. Figure 3c shows the tree structure detected by VISDA. The clusters at the leaf nodes of the hierarchy form the final clustering result. For the colon cancer cluster (the third cluster at the third level of the hierarchy), the one sub-cluster model and the two sub-cluster model have a description length of 1446.59 and 1445.50, respectively, which are very close. By examining the two sub-cluster partition, we find that one of the sub-clusters essentially only contains the two left-most samples in the cluster, which are apparently outliers. Thus we choose not to decompose this cluster.

## Results

### Evaluation of VISDA

In a comparative study of clustering algorithms [23], we evaluated VISDA by comparing it to four other popular unsupervised clustering methods – conventional HC, KMC, sequential SOM, and SFNM fitting methods. The comparison was made with respect to clustering accuracy and stability, evaluated on one synthetic dataset and seven microarray gene expression datasets described in Table 1 of Additional file 1. For a meaningful and well-grounded evaluation, we directly compared the sample clustering results to the ground-truth biological categories for measurement of algorithm performance. To assure the quality and suitability of the datasets with respect to the definitive ground truth for a rigorous and fair comparison, the datasets were preprocessed by a supervised informative gene selection method introduced in [58]. The preprocessed datasets covered both the "data-sufficient" case and the "data-insufficient" case, the latter having a small samples-to-genes ratio. The experiment was conducted based on *n*-fold cross-validation (*n *equal to 9 or 10 depending on the sample size), i.e. in each trial, only an (*n*-1)/*n *portion of the samples from each class were used in the model learning.

**Table 1:** Comparison of clustering performance

	VISDA	HC	KMC	SOM (MSC)	SOM (CLL)	SFNM Fitting
Average mean of partition accuracy	**86.29%**	58.89%	76.47%	76.52%	79.39%	64.47%
Average standard deviation of partition accuracy	4.01%	5.03%	3.92%	**3.85%**	4.73%	5.07%

The bolded font indicates the best performance respective to a particular measure.

Because the clustering results of KMC, SOM and SFNM fitting methods may depend on initialization, for each cross-validation trial we ran them 100 times with random initialization and took the best clustering result according to the associated optimization criterion. For KMC, since its algorithm tries to minimize Mean Squared Compactness (MSC), which is the average square distance from each data point to its cluster center, we selected the result with the minimum MSC. For SOM, we separately tried minimizing MSC and maximizing Classification Log-Likelihood (CLL) [23], which calculates the log-likelihood of the model by assigning each cluster a Gaussian distribution whose parameters are calculated based on the samples within the cluster. Not like the soft memberships in mixture modeling, CLL calculates the log-likelihood in a "hard" manner, where each sample is generated only by the Gaussian distribution of its cluster. For SFNM fitting, since the algorithm tries to maximize the likelihood of the SFNM model, we selected the result with the maximum likelihood. HC was applied with Euclidean distance and average linkage function. For HC, KMC, SOM, and SFNM fitting method, we set the input cluster number at the number of ground-truth classes. Because VISDA does not utilize any information about the number of classes and class labels of the samples, VISDA obtained the cluster number and clustering partition in a purely unsupervised fashion. We calculated the frequency that VISDA obtained the correct cluster number (taken as the number of ground-truth classes in the dataset) across the cross-validation trials to evaluate its model selection accuracy. The partition accuracy, i.e. the percentage of correctly labeled samples after clustering, was used to evaluate the biological relevance of the obtained clusters. For soft clustering, to calculate the partition accuracy, we transformed the soft memberships to hard memberships via the MAP rule. The mean partition accuracy of all cross-validation trials was calculated and then averaged over all datasets (shown in Table 1). We also calculated the standard deviation of the partition accuracy resulted from cross-validation to see how stable the biological relevance was. The standard deviation of the partition accuracy was also averaged over all datasets and shown in Table 1.

VISDA obtained the correct cluster number across the cross-validation trials with an average frequency of 97% over all the datasets. This shows that the exploration based on the hierarchical SFNM model, MDL model selection in the locally discriminative visualization space, and human-data interaction for data visualization, model initialization, and model validation, is an effective solution for model selection working on high dimensional data. VISDA outperformed all other methods in average mean of partition accuracy, which shows that VISDA's clustering result was the most accurate among the competing methods. From the average standard deviation of partition accuracy, we can see that VISDA is also a stable performer among the competing methods. Besides partition accuracy, we also evaluated the accuracy of the recovered parametric class distributions (the first and second order statistics), where the result is similar [23], i.e. VISDA provides a stable and most accurate recovery of class distributions among the competing methods.

### Identification of gene clusters from muscular dystrophy data and muscle regeneration data

**Figure 4:**
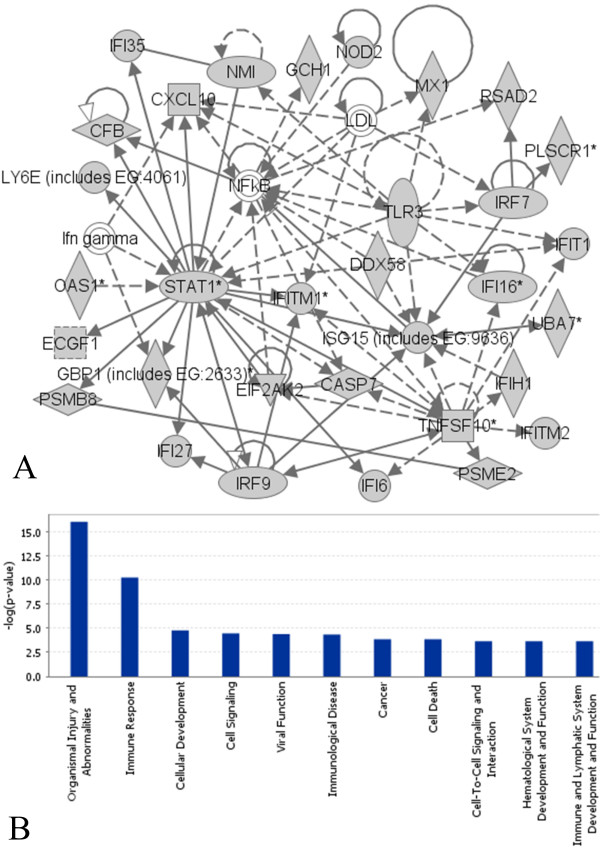
**Analysis results of the detected gene cluster**. (a) Top scoring gene regulation network indicated by the gene cluster. Grey colour indicates that the gene is in the detected gene cluster. Solid lines indicate direct interactions. Dashed lines indicate indirect interactions. (b) The negative log *p*-values of the most significant functional categories associated with the gene cluster. These two figures are from the IPA system.

On a muscular dystrophy microarray gene expression dataset (Table 2 in Additional file 1 gives a brief description of the dataset) [6], we used VISDA to define gene clusters to discover functional gene groups and gene regulation networks. After performing the clustering, we superimposed existing knowledge of gene regulation and gene function from Ingenuity Pathway Analysis database (IPA, ) to analyze some of the obtained clusters that had interesting expression value patterns. We found that one gene cluster that contained 122 genes was highly expressed in Juvenile DermatoMyositis (JDM) but lowly expressed in all other phenotypes. JDM is a relatively severe childhood autoimmune disorder that is thought to be associated with viral infections that stimulate muscle destruction by inflammatory cells and ischemic processes in a small subset of the children with the virus. IPA showed that this gene cluster pointed to gene regulatory networks involved in key inflammatory pathways. In the most significant network (with a negative log *p*-value of 77), shown in Figure 4a, specific proteins that are known to be critical for initiating and perpetuating inflammation and subsequent cell death are seen as key focus genes (IPA calculated the *p*-values based on the hypergeometric distribution, via Fisher's exact test for 2 × 2 contingency tables.). STAT1 is an important signaling molecule that responds to interferons and other cytokines. Both TNFSF10 and CASP7 influence cell death via apoptosis. Consistent with this, patients with JDM show extensive cell death and failure of regeneration in their muscle, leading to weakness. This network also points to drugs that would be expected to inhibit this process in JDM patients, which can be tested in a mouse model. Figure 4b shows that this gene cluster was significantly associated with organismal injury/abnormalities and immune response in terms of gene function category.

In another study [46], we used VISDA to define gene clusters in a 27 time point microarray gene expression dataset of muscle regeneration in vivo based on the mouse model. After pre-filtering by "present call", 7570 genes were believed to be significantly present and were thus input to VISDA for gene clustering. Two of the eighteen gene clusters detected by VISDA peaked at the 3rd day time point, which correlated with the expression pattern of MyoD, a prototypical member of myogenic regulatory factors that control the staged induction of genes important for interpretation of positional cues, proliferation, and differentiation of myogenic cells. These two clusters contained a subset of the in vitro MyoD down-stream targets identified in [59], which characterized the relevance of in vitro myogenesis to in vivo muscle regeneration.

### Construction of TOPs on muscular dystrophy data and multi-category cancer data

**Figure 5:**
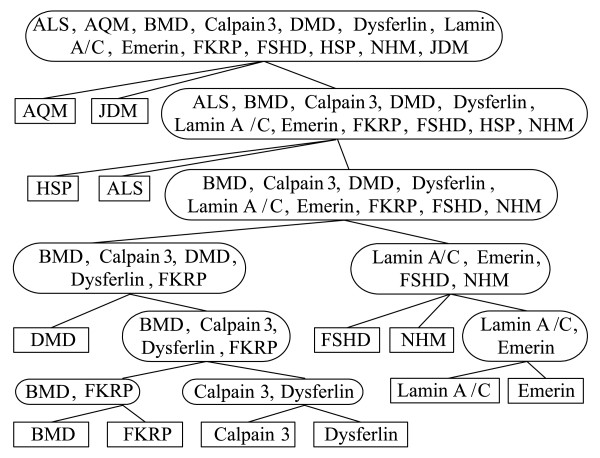
**The TOP found by VISDA on the muscular dystrophy dataset**. Rectangles contain individual phenotypes. Ellipses contain a group of phenotypes.

We used VISDA to cluster phenotypes in the muscular dystrophy dataset (Table 2 in Additional file 1 gives a brief introduction of the dataset) [6]. The dataset includes 13 muscular dystrophy related phenotypes, i.e. ALS, AQM, BMD, Calpain3, DMD, Dysferlin, Lamin A/C, Emerin, FKRP, FSHD, HSP, JDM, and NHM [6]. The TOP constructed by VISDA with *n*_*g *_(the number of selected genes per phenotype) equal to 2 is shown in Figure 5. AQM, JDM, ALS, and HSP were first separated from the rest, which is consistent with each of them having an underlying disease mechanism much different from the other classes. Then, the tree showed two major branches. The left branch contained BMD, Calpain 3, DMD, Dysferlin, and FKRP, most of which are the "dystrophic myopathies", inborn single gene disorders causing degeneration/regeneration of muscle fibers. The right branch contained Lamin A/C, Emerin, FSHD, and NHM. The two nuclear envelope disorders, Lamin A/C and Emerin, form their own group, showing their close relationship reflected at mRNA profiles. FSHD disrupts chromatin attachment sites to the nuclear envelope, which supports its co-segregation with Lamin A/C and Emerin in the right branch.

On the 14 class MIT microarray gene expression cancer dataset with 190 samples (Table 3 in Additional file 1 gives a brief description of the dataset) [24,60], we applied leave-one-out stability analysis with *n*_*g *_equal to 6. In each experimental trial of the leave-one-out stability analysis, one sample was left out and we constructed a TOP based on the remaining samples. Thus totally 190 TOPs were generated and we took the tree with the highest frequency of occurrence as the final solution, which best reflects the underlying stable structure of the data. As a validation, we compared the most frequent TOP to the known developmental/morphological relationships among the various cancer classes, which was published in [60].

**Figure 6:**
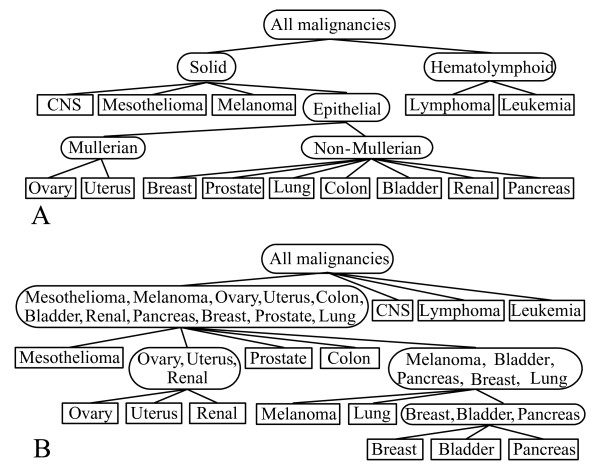
**Comparison between the most frequent TOP and the pathological relationships among the cancer classes**. (a) Published developmental/morphological relationships among the cancer classes. (b) The most frequent TOP constructed by VISDA. Rectangles contain one cancer type. Ellipses contain a group of cancers.

Forty three different TOPs occurred in the leave-one-out stability analysis. The most frequent TOP occurred 121 times; the second most frequent TOP occurred 11 times; the third most frequent TOP occurred 7 times; most of the other TOPs only occurred once. The most frequent TOP has an occurrence frequency of 121/190 ≈ 63.68%. Considering that some TOPs have only minor differences compared to the most frequent TOP, the underlying stable structure likely has even a higher occurrence frequency. We also applied VISDA on the whole dataset and obtained the same structure as the most frequent TOP. Figure 6a shows the known pathological cancer tree [60] and Figure 6b shows the obtained most frequent TOP. We can see that the most frequent TOP captured some pathological relationships reflected in mRNA profiles. The neoplasm of lymphoma and leukemia are hematolymphoid; appropriately, in the most frequent TOP, they were far away from the other cancer classes whose neoplasm is solid. CNS and mesothelioma were separated from epithelial tumors. The ovary cancer and the uterus cancer are mullerian tumors and closely located in the tree. Breast, bladder and pancreas cancer belong to the non-mullerian category and formed a tight subgroup.

## Discussion

VISDA is a data analysis tool incorporating human intelligence and domain knowledge. When applied by experienced users and domain experts, VISDA is more likely to generate accurate/meaningful clustering and visualization results. Since different human-data interaction may lead to different clustering outcomes, to achieve optimum performance, the user needs to acquire experience in using VISDA on various kinds of data, especially on the dataset of interest. Multiple trials applying VISDA are suggested when analyzing a new dataset. By comparing VISDA to several popular clustering methods, we see that the clustering outcome of VISDA is stable, probably because human initialization of model parameters has the potential to improve the clustering stability compared to the random parameter initialization applied by some other methods. Notice that VISDA only requires the user to have common sense about cluster distributions, cluster separability, and outliers.

Besides the two kinds of non-informative genes discussed in the Methods section, "redundant" genes (genes that are highly correlated with other genes) provide only limited additional separability between sample clusters. However, this limited additional separability may in fact greatly improve the achievable partition accuracy [61]. Thus, we take removal of redundant genes as an optional step for sample clustering. If the dimensionality of the gene space after variation filtering and discrimination power filtering can not be well handled by the clustering algorithm (i.e. if the samples-to-genes ratio is not sufficiently large), we suggest removing highly correlated genes. Here, we provide a simple scheme to remove redundant genes. In the gene list resulting from variation filtering and discrimination power analysis, keep the most discriminative gene and remove the genes that are highly correlated with it. Then keep the second most discriminative gene in the remaining list and remove the genes that are highly correlated with this second most discriminative gene. Keep performing this procedure until no further removal can be done. The correlation between genes can be measured by Pearson correlation coefficient or mutual information normalized by entropy. A threshold needs to be set to identify the highly correlated genes.

Various visualization techniques, such as dendrogram, heat maps, and projections, have been applied to present genomic data structures and clustering outcomes [2,16,62,63]. Many linear/nonlinear projection methods, such as PCA [22], random projection [33], variant of multi-dimensional scaling [62], and projection based on frequency domain analysis [63], have been used to visualize/analyze genomic data. In VISDA, data are hierarchically visualized using multiple local data projections, one at each node of the hierarchy. Such a hierarchical visualization scheme allows each local data projection to be fulfilled by relatively simple method, i.e. linear projection, while the whole visualization hierarchy is capable to reveal both global and local cluster structures. Since every clustering and visualization method has its own underlying assumptions about the cluster structure of interest [8-10,39-41], VISDA provides users with an extensible visualization capability by a projection suite that can incorporate novel, effective, complementary projection methods to increase the likelihood of revealing the data/application-dependent cluster structure of interest. Besides enhancing human understanding of the data structure, data visualization in VISDA has a further function of providing the basis for introducing human intelligence and domain knowledge to the clustering process.

One point needs to be noted is that VISDA selects a data model in the locally discriminative low dimensional visualization space. Although visualization with dimension reduction may reveal only the main data structure and lose minor/local data structures within a cluster, these minor/local structures may become the main data structure captured at subsequent levels. VISDA discovers hierarchical relationships between clusters, which allows analyzing the data at different resolutions/scales.

Larger clusters can be obtained by simply merging small clusters according to the hierarchy. The discovered hierarchical relationships among clusters may reveal important biological information, for example the developmental/morphological information revealed by TOPs. The TOP discovered by VISDA can also be used to construct a hierarchical classifier to solve the complex task of multiple diseases diagnosis by embedding a relatively simple classifier at each node of the TOP, which may obtain good classification performance [64].

Despite our successful applications of VISDA to real microarray gene expression data, there are remaining limitations of the reported method. For example, in sample clustering, dimension reduction via unsupervised informative gene selection is highly data-dependent and often achieves only limited success. This is a very challenging task due to no prior knowledge and potentially complex gene-gene interactions embedded within high dimensional data. Furthermore, user-data interaction may bring certain subjectivity into the clustering process if not being properly orchestrated, and projection visualization may cause some unrecoverable information loss leading to only a suboptimum solution, although VISDA's hierarchical framework can partially alleviate this problem. Lastly, VISDA presently assumes each cluster follows a Gaussian distribution largely driven by mathematical convenience. However, small sample size problem can defeat this assumption and composite clusters at higher-levels of the hierarchy are not even theoretically normally distributed but are more generally mixture distributions.

Our previous publications [23,44] may also help practitioners in using VISDA for genomic data analysis. Reference [44], the caBIG™ VISDA application note, is a brief and fast-track "software user guide" that provides users with information on the procedures and interfaces of caBIG™ VISDA open-source software. Reference [23] focuses on a comparative experiment of several clustering algorithms including VISDA to study their relative performance and suitability for genomic data clustering. Although these two papers do not discuss the principles/algorithms of VISDA, they are helpful to readers interested in using VISDA for practical tasks of genomic data analysis.

## Conclusion

We designed a clustering and visualization algorithm for discovering structure in high dimensional genomic data. VISDA can discover and visualize gene clusters, sample clusters, phenotype clusters, and the hierarchical relationships between the detected clusters. VISDA visualizes data by structure-preserving projections and provides an interface for human-data interaction, which facilitates incorporation of expert domain knowledge and human intelligence to help achieve accurate and meaningful data visualization and modeling. The scalable and extensible VISDA framework can incorporate various existing clustering and visualization algorithms to increase the likelihood of revealing data structure of interest.

Our evaluation study based on microarray gene expression data showed that VISDA provided an effective model selection scheme for high dimensional data and outperformed several popular clustering methods, i.e. HC, KMC, SOM, and SFNM fitting, in terms of clustering accuracy. Applications to muscular dystrophy, muscle regeneration, and cancer data illustrated that VISDA produced biologically meaningful clustering results that can enhance users' understanding about the underlying biological mechanism and stimulate novel hypotheses for further research.

## Abbreviations

VISDA: VIsual Statistical Data Analyzer; SOM: Self-Organizing Maps; KMC: K-Means Clustering; HC: conventional Hierarchical Clustering; MDL: Minimum Description Length; TOP: Tree Of Phenotypes; SFNM: Standard Finite Normal Mixture; PCA: Principal Component Analysis; PCA-PPM: Principal Component Analysis – Projection Pursuit Method; LPP: Locality Preserving Projection; DCA: Discriminatory Component Analysis; APC: Affinity Propagation Clustering; EM algorithm: Expectation Maximization algorithm; MSC: Mean Squared Compactness; CLL: Classification Log-Likelihood; IPA: Ingenuity Pathway Analysis; JDM: Juvenile DermatoMyositis.

## Authors' contributions

YZ participated in designing and implementing VISDA, performing the experiment, and analyzing the experimental results. HL participated in developing caBIG™ VISDA. DJM participated in the technical design of VISDA on phenotype clustering. ZW implemented the prototype of VISDA. JX participated in the technical design of the software and comparative study. RC and EPH provided biological interpretation of the datasets and experimental results. YW participated in designing VISDA and the experiment, and provided technical supervision.

## Supplementary Material

Additional file 1 **caBIG™ VISDA: modeling, visualization, and discovery for cluster analysis of genomic data (supplement).** The supplement includes derivations and details of the algorithm, more discussions, and introduction of the datasets used in the experiments. Click here for file

## References

[B1] Golub TR, Slonim DK, Tamayo P, Huard C, Gaasenbeek M, Mesirov JP, Coller H, Loh ML, Downing JR, Caligiuri MA (1999). Molecular classification of cancer: class discovery and class prediction by gene expression monitoring. Science.

[B2] Eisen MB, Spellman PT, Brown PO, Botstein D (1998). Cluster analysis and display of genome-wide expression patterns. Proc Natl Acad Sci USA.

[B3] Gong T, Xuan J, Wang C, Li H, Hoffman EP, Clarke R, Wang Y (2007). Gene module identification from microarray data using nonnegative independent component analysis. Gene Regulation and Systems Biology.

[B4] Wu CJ, Fu Y, Murali TM, Kasif S (2004). Gene expression module discovery using gibbs sampling. Genome Inform.

[B5] Miller DJ, Wang Y, Kesidis G (2008). Emergent unsupervised clustering paradigms with potential application to bioinformatics. Front Biosci.

[B6] Bakay M, Wang Z, Melcon G, Schiltz L, Xuan J, Zhao P, Sartorelli V, Seo J, Pegoraro E, Angelini C (2006). Nuclear envelope dystrophies show a transcriptional fingerprint suggesting disruption of Rb-MyoD pathways in muscle regeneration. Brain.

[B7] Zhu Y, Wang Z, Feng Y, Xuan J, Miller DJ, Hoffman EP, Wang Y (2006). Phenotypic-specific gene module discovery using a diagnostic tree and caBIG™ VISDA. Proc IEEE Int Conf EMBS: New York City.

[B8] Jain AK, Murty MN, Flynn PJ (1999). Data clustering: a review. ACM Comp Surv.

[B9] Jiang D, Tang C, Zhang A (2004). Cluster analysis for gene expression data: a survey. IEEE Trans Know Data Eng.

[B10] Xu R, Wunsch D (2005). Survey of clustering algorithms. IEEE Trans Neural Networks.

[B11] Yeung KY, Fraley C, Murua A, Raftery AE, Ruzzo WL (2001). Model-based clustering and data transformations for gene expression data. Bioinformatics.

[B12] Pan W (2006). Incorporating gene functions as priors in model-based clustering of microarray gene expression data. Bioinformatics.

[B13] Roth V, Lange T (2004). Bayesian class discovery in microarray datasets. IEEE Trans Biomed Eng.

[B14] Huttenhower C, Flamholz A, Landis J, Sahi S, Myers C, Olszewski K, Hibbs M, Siemers N, Troyanskaya O, Coller H (2007). Nearest neighbor networks: clustering expression data based on gene neighborhoods. BMC Bioinformatics.

[B15] Ben-Dor A, Shamir R, Yakhini Z (1999). Clustering Gene Expression Patterns. J Comput Biol.

[B16] Monti S, Tamayo P, Mesirov J, Golub T (2003). Consensus clustering: a resampling-based method for class discovery and visualization of gene expression microarray data. Mach Learn.

[B17] Tamayo P, Slonim D, Mesirov J, Zhu Q, Kitareewan S, Dmitrovsky E, Lander ES, Golub TR (1999). Interpreting patterns of gene expression with self-organizing maps: methods and application to hematopoietic differentiation. Proc Natl Acad Sci USA.

[B18] Tavazoie S, Hughes JD, Campbell MJ, Cho RJ, Church GM (1999). Systematic determination of genetic network architecture. Nature Genet.

[B19] Dembele D, Kastner P (2003). Fuzzy C-means method for clustering microarray data. Bioinformatics.

[B20] Fu L, Medico E (2007). FLAME, a novel fuzzy clustering method for the analysis of DNA microarray data. BMC Bioinformatics.

[B21] Bishop CM (1995). Neural Networks for Pattern Recognition.

[B22] Duda RO, Hart PE, Stork DG (2001). Pattern Classification.

[B23] Zhu Y, Wang Z, Miller DJ, Clarke R, Xuan J, Hoffman EP, Wang Y (2008). A ground truth based comparative study on clustering of gene expression data. Front Biosci.

[B24] Ramaswamy S, Tamayo P, Rifkin R, Mukherjee S, Yeang C, Angelo M, Ladd C, Reich M, Latulippe E, Mesirov JP (2001). Multiclass cancer diagnosis using tumor gene expression signatures. Proc Natl Acad Sci USA.

[B25] Clarke R, Ressom HW, Wang A, Xuan J, Liu MC, Gehan EA, Wang Y (2008). The properties of high-dimensional data spaces: implications for exploring gene and protein expression data. Nat Rev Cancer.

[B26] Datta S, Datta S (2006). Methods for evaluating clustering algorithm for gene expression data using a reference set of functional classes. BMC Bioinformatics.

[B27] McShane LM, Radmacher MD, Freidlin B, Yu R, Li M-C, Simon R (2002). Methods for assessing reproducibility of clustering patterns observed in analyses of microarray data. Bioinformatics.

[B28] Smolkin M, Ghosh D (2003). Cluster stability scores for microarray data in cancer studies. BMC Bioinformatics.

[B29] Rissanen J (1978). Modeling by shortest data description. Automatica.

[B30] Schwarz G (1978). Estimating the dimension of a model. Ann Statistics.

[B31] Graham MW, Miller DJ (2006). Unsupervised learning of parsimonious mixtures on large spaces with integrated feature and component selection. IEEE Trans Signal Process.

[B32] Bertoni A, Valentini G (2007). Model order selection for bio-molecular data clustering. BMC Bioinformatics.

[B33] Bertoni A, Valentini G (2008). Discovering multi-level structures in bio-molecular data through the Bernstein inequality. BMC Bioinformatics.

[B34] Lange T, Roth V, Braun ML, Buhmann JM (2004). Stability-based validation of clustering solutions. Neural Comput.

[B35] Wang Y, Miller DJ, Clarke R (2008). Approaches to working in high dimensional data spaces: gene expression microarray. Brit J Cancer.

[B36] Xing EP, Karp RM (2001). CLIFF: clustering of high-dimensional microarray data via iterative feature filtering using normalized cuts. Bioinformatics.

[B37] Brown MPS, Grundy WN, Lin D, Cristianini N, Sugnet C, Furey TS, Ares M, Haussler D (2000). Knowledge-based analysis of microarray gene expression data using support vector machines. Proc Natl Acad Sci USA.

[B38] Qu Y, Xu S (2004). Supervised cluster analysis for microarray data based on multivariate Gaussian mixture. Bioinformatics.

[B39] Chien Y (1978). Interactive Pattern Recognition.

[B40] Zou J, Nagy G, Basu M, Ho TK (2006). Human-computer interaction for complex pattern recognition problems. Data complexity in pattern recognition.

[B41] Bishop CM, Tipping ME (1998). A hierarchical latent variable model for data visualization. IEEE Trans Pattern Anal Mach Intell.

[B42] Tipping M, Bishop C (1999). Mixtures of probabilistic principal component analyzers. Neural Comput.

[B43] Wang Y, Luo L, Freedman MT, Kung S (2000). Probabilistic principal component subspaces: a hierarchical finite mixture model for data visualization. IEEE Trans Neural Networks.

[B44] Wang J, Li H, Zhu Y, Yousef M, Nebozhyn M, Showe M, Showe L, Xuan J, Clarke R, Wang Y (2007). VISDA: an open-source caBIG™ analytical tool for data clustering and beyond. Bioinformatics.

[B45] Wang Z, Wang Y, Lu J, Kung S, Zhang J, Lee R, Xuan J, Khan J, Clarke R (2003). Discriminatory mining of gene expression microarray data. J VLSI Signal Proces.

[B46] Zhao P, Seo J, Wang Z, Wang Y, Shneiderman B, Hoffman EP (2003). In vivo filtering of in vitro expression data reveals MyoD targets. C R Biol.

[B47] Hyvärinen A, Karhunen J, Oja E (2001). Independent Component Analysis.

[B48] He X, Niyogi P, Thrun S, Saul LK, Schölkopf B (2004). Locality preserving projections. Advances in Neural Information Processing Systems 16.

[B49] Meyer CD (2000). Matrix analysis and applied linear algebra. SIAM.

[B50] Loog M, Duin RPW, Haeb-Umbach R (2001). Multiclass linear dimension reduction by weighted pairwise fisher criteria. IEEE Trans Pattern Anal Mach Intell.

[B51] Frey BJ, Dueck D (2007). Clustering by passing messages between data points. Science.

[B52] Weiss Y, Freeman WT (2001). On the optimality of solutions of the max-product belief-propagation algorithm in arbitrary graphs. IEEE Trans Inform Theory.

[B53] Dempster AP, Laird NM, Rubin DB (1977). Maximum likelihood from incomplete data via the EM algorithm. J R Statist Soc, Series B.

[B54] Ridder FD, Pintelon R, Schoukens J, Gillikin DP (2005). Modified AIC and MDL model selection criteria for short data records. IEEE Trans Instrum Meas.

[B55] Liang Z, Jaszczak RJ, Coleman RE (1992). Parameter estimation of finite mixtures using the EM algorithm and information criteria with application to medical image processing. IEEE Trans Nucl Sci.

[B56] Miller DJ, Browning J (2003). A mixture model and EM-based algorithm for class discovery, robust classification, and outlier rejection in mixed labeled/unlabeled data sets. IEEE Trans Pattern Anal Mach Intell.

[B57] Giordano TJ, Shedden KA, Schwartz DR, Kuick R, Taylor JMG, Lee N, Misek DE, Greenson JK, Kardia SLR, Beer DG (2001). Organ-specific molecular classification of primary lung, colon, and ovarian adenocarcinomas using gene expression profiles. Am J Pathol.

[B58] Xuan J, Wang Y, Dong Y, Feng Y, Wang B, Khan J, Bakay M, Wang Z, Pachman L, Winokur S (2007). Gene selection for multiclass prediction by weighted fisher criterion. EURASIP J Bioinform and Syst Biol.

[B59] Bergstrom DA, Penn BH, Strand A, Perry RL, Rudnicki MA, Tapscott SJ (2002). Promoter-specific regulation of MyoD binding and signal transduction cooperate to pattern gene experssion. Mol Cell.

[B60] Shedden KA, Taylor JM, Giordano TJ, Kuick R, Misek DE, Rennert G, Schwartz DR, Gruber SB, Logsdon C, Simeone D (2003). Accurate molecular classification of human cancers based on gene expression using a simple classifier with a pathological tree-based framework. Am J Pathol.

[B61] Guyon I, Elisseeff A (2003). An introduction to variable and feature selection. J Mach Learn Res.

[B62] Ewing RM, Cherry JM (2001). Visualization of expression clusters using Sammon's non-linear mapping. Bioinformatics.

[B63] Zhang L, Zhang A, Ramanathan M (2004). VizStruct: exploratory visualization for gene expression profiling. Bioinformatics.

[B64] Feng Y, Wang Z, Zhu Y, Xuan J, Miller D, Clarke R, Hoffman E, Wang Y (2006). Learning the tree of phenotypes using genomic data and VISDA. Proc IEEE Symp Bioinform and Bioeng: Arlington, VA, USA.

