# Supplementary material for: caBIG™ VISDA: Modeling, visualization, and discovery for cluster analysis of genomic data
Source: BMC Bioinformatics. 2008 Sep 18;9:383. doi: 10.1186/1471-2105-9-383 (PMC2566986; doi:10.1186/1471-2105-9-383)
Supplement: Additional file 1 — caBIG™ VISDA: modeling, visualization, and discovery for cluster analysis of genomic data (supplement). The supplement includes derivations and details of the algorithm, more discussions, and introduction of the datasets used in the experiments. [file 1471-2105-9-383-S1.pdf]

caBIG^{TM} VISDA: modeling, visualization, and discovery for cluster analysis of genomic data (supplement)

Yitan Zhu[1], Huai Li[1,2], David J. Miller[3], Zuyi Wang[1,4], Jianhua Xuan[1], Robert Clarke[5], Eric P. Hoffman[4], and Yue Wang[1]

[1]Department of Electrical and Computer Engineering, Virginia Polytechnic and State University, Arlington, VA 22203, USA
[2]Bioinformatics Unit, RRB, National Institute on Aging, NIH, Baltimore, MD 21224, USA
[3]Department of Electrical Engineering, The Pennsylvania State University, University Park, PA 16802, USA
[4]Research Center for Genetic Medicine, Children's National Medical Center, Washington, DC 20010, USA
[5]Department of Oncology, Physiology & Biophysics and Lombardi Comprehensive Cancer Center, Georgetown University, Washington, DC 20007, USA

1. LIMITATIONS OF EXISTING ALGORITHM

1.1 Initialization sensitivity, local optimum and solution reproducibility

There are multiple local optimums of partitional clustering objective functions such as KMC and mixture modeling. Standard learning methods optimize the objective function and thus find the local optimum solution "nearest to'' the model initialization. More sophisticated methods or initialization schemes, which seek to avoid poor local optimums, exist, but require significant computation and normally do not ensure convergence to the global optimum (Rose, 1998). The quality of the local optimum may be decidedly poor, compared to that of the global optimum. This is especially true for genomic data sets, with high dimensionality and small sample size (Bishop, 1995; Duda, *et al*., 2001; Zhu, *et al*., 2008). On the other hand, methods such as conventional agglomerative Hierarchical Clustering (HC) perform a very simple, greedy optimization, which severely limits the amount of search they perform over the space of possible solutions, and thus limits the solution accuracy.

1.2 Solution reproducibility

Since clustering may help drive scientific hypotheses, it is extremely important that solutions be reproducible/robust. This refers to the ability of a clustering algorithm to identify the same (or quite similar) structure under different model initializations, in the presence of small data set perturbations or additive noise, as well as when analyzing independent data sets drawn from the same underlying distribution. KMC and mixture modeling, which are sensitive to model initialization, do not yield reproducible solutions when random model initialization is applied. However, more sophisticated initialization, e.g., applying the splitting algorithm (Hartigan, 1975) to initialize KMC cluster centers, will yield less variable KMC solutions. Likewise, robust KMC solutions can in turn be used to provide a robust initialization for mixture model fitting. On the other hand, agglomerative HC (e.g., the single linkage algorithm (Duda, *et al*., 2001)) is highly sensitive to the particular data sample and to the noise in the data.

1.3 Order selection: estimating the number of clusters

For hierarchical clustering, simple heuristics are typically applied to estimate the number of clusters, such as identifying the order at which a large improvement in fitting accuracy occurs. For mixture modeling, information-theoretic model selection criteria such as minimum description length (MDL) are often applied. These criteria consist of a data fitting term (mismatch between data and model) and a model complexity term. For sample clustering on genomic data, with high feature/gene dimensionality and small sample size, use of such criteria is likely to grossly underestimate the number of clusters because each gene will entail at least one free parameter per cluster, leading to a very high complexity penalty when increasing the number of clusters. This can be mitigated by front-end gene selection or embedding feature selection within clustering (Graham and Miller, 2006; Wang, *et al*., 2008), which reduce the gene number and, thus, model complexity, for a given number of clusters.

1.4 Unsupervised feature selection

Unsupervised informative gene selection for sample clustering is a critical yet difficult problem due to the existence of many irrelevant genes respective to the phenotypes/sub-phenotypes of interest (Jiang, *et al*., 2004; Xu and Wunsch, 2005) – without first possessing correct clusters, it is difficult to identify relevant genes, and without good gene selection to eliminate many noisy/irrelevant ones, it is very difficult to discern the true underlying cluster structure. Iterative algorithms, which embed gene selection within sample clustering to bootstrap this problem, have been developed and tested only for the two-cluster case (Roth and Lange, 2004; Xing and Karp, 2001). There is also work on this problem from the machine learning community, e.g. (Dy and Brodley, 2000; Graham and Miller, 2006) that is not limited to the two-cluster case. However, more research effort specifically targeting genomic data is needed.

1.5 Confounding variables and multiple sources of cluster structure

A fundamental, flawed assumption in much clustering work is that there is a single source of clustering tendency in the data – e.g., “disease’’ vs. “non-disease’’. There may be other sources of clustering tendency, based either on measured or even unmeasured (latent) factors, e.g. gender, environment, measurement platform (Benito, *et al*., 2004), or other biological processes that are peripheral or at any rate not of direct interest in the current study (Clarke, *et al*., 2008). While there is some research in this area (Benito, *et al*., 2004), more work is needed in removing or accounting for confounding influences in genomics research (Miller, *et al*., 2008; Wang, *et al*., 2008).

1.6 Exploitation of prior knowledge and human interaction

The most fundamental limitation in clustering is the ill-posed nature of the problem. The number of clusters depends on the scale at which one views the data (Rose, 1998) and the data grouping clearly depends on the geometric or statistical assumption/definition of cluster (Duda, *et al*., 2001; Lange, *et al*., 2004). Even when there is a known parametric mixture model form, there may be no unique solution, i.e. there is the mixture identifiability problem (Duda, *et al*., 2001). What can break nonuniqueness and ill-posedness is to incorporate prior knowledge. Incorporating prior knowledge may also help to focus the clustering analysis on interesting data structure and remove the effect of confounding variables. Unfortunately, most clustering algorithms do not have mechanisms for exploiting prior information – exceptions include semi-supervised gene clustering methods that utilize gene annotations to form gene clusters (Brown, *et al*., 2000; Pan, 2006; Qu and Xu, 2004) and some other more general semi-supervised methods (Zhu, 2006). Besides auxiliary database information, the user’s (expert’s) domain knowledge and the human gifts for pattern recognition and data visualization in low-dimensional spaces can also help to produce accurate and meaningful clustering outcomes (Chien, 1978; Zou and Nagy, 2006). For example, Bishop and Tipping developed a hierarchical data visualization and exploration scheme based on a mixture of latent variables model with human-data interaction (Bishop and Tipping, 1998; Tipping and Bishop, 1999).

1.7 Inflexibility and non-adaptivity of standard methods

Most clustering algorithms have a standalone nature and make underlying statistical or geometric assumptions about clusters. When these assumptions are violated, the method may fail badly, and with no “backup plan’’, i.e. no capability to modify the assumptions to seek a better result. A recent strategy with some ability to mitigate this is ensemble or consensus clustering (Strehl and Ghosh, 2002; Topchy, *et al*., 2005), wherein multiple algorithms are applied and their results then fused so as to maximize a clustering “consensus’’ function. However, these methods, again being fully automated, cannot benefit from human interaction and expert knowledge, which can guide algorithm choices in a flexible, adaptive manner, to match the underlying data characteristics and the (domain-specific) clustering structure of interest. In particular, as demonstrated in this paper, clustering and data visualization can complement each other, providing a “transparent’’ clustering process that enhances the user’s understanding of the data and that informs clustering via choices based on expert knowledge and human intelligence.

2. METHOD

2.1 Algorithm for gene clustering and sample clustering

Let $\mathbf{t} = \{\mathbf{t}_1, \mathbf{t}_2, \ldots, \mathbf{t}_N \mid \mathbf{t}_i \in \mathrm{R}^p, i = 1, 2, \ldots, N\}$ denote N p-dimensional data points to be clustered. Suppose that the hierarchical exploration proceeds to the lth level, i.e. K_l clusters are detected at level l and the posterior probability of data point $\mathbf{x}_i$ belonging to cluster k ($k = 1, \ldots, K_l$) is $z_{i,k}$.

2.1.1 Visualization by complementary structure-preserving projections

For cluster k, VISDA projects the cluster onto 2-D spaces by five projection methods, Principal Component Analysis (PCA), Principal Component Analysis – Projection Pursuit Method (PCA–PPM) (Wang, *et al.*, 2003), HC–KMC–SFNM–DCA (Zhu, *et al.*, 2006), Locality Preserving Projection (LPP) (He and Niyogi, 2004), and APC–DCA, where SFNM refers to Standard Finite Normal Mixture, DCA refers to Discriminative Component Analysis and APC refers to Affinity Propagation Clustering (Frey and Dueck, 2007).

PCA uses the eigenvectors associated with the largest eigenvalues of the cluster's covariance matrix as projection directions. The covariance matrix of cluster k is calculated by

$$\begin{aligned} \boldsymbol{\mu}_{\mathbf{t},k} &= \sum_{i=1}^{N} z_{i,k}\mathbf{t}_i \Big/ \sum_{i=1}^{N} z_{i,k} \\ \boldsymbol{\Sigma}_{\mathbf{t},k} &= \sum_{i=1}^{N} z_{i,k}\left(\mathbf{t}_i - \boldsymbol{\mu}_{\mathbf{t},k}\right)\left(\mathbf{t}_i - \boldsymbol{\mu}_{\mathbf{t},k}\right)^T \Big/ \sum_{i=1}^{N} z_{i,k} \end{aligned}, \qquad (1)$$

where $\boldsymbol{\mu}_{\mathbf{t},k}$ is the mean of cluster k, $\boldsymbol{\Sigma}_{\mathbf{t},k}$ is the covariance matrix of cluster k. The subscript '**t**' indicates that these parameters model the data in the original data space.

PCA–PPM selects two of the eigenvectors on which the projected data distribution has the smallest kurtosis, because flat distributions or distributions with thick tails usually reveal structure information and kurtosis measures the peakedness of a distribution (Hyvärinen*, et al.*, 2001). For any projection vector $\mathbf{w}$, the kurtosis of the projected data distribution is calculated by

$$y_i = \mathbf{w}^T \times \left(\mathbf{t}_i - \boldsymbol{\mu}_{\mathbf{t},k}\right)$$

$$kurtosis = \frac{\sum_{i=1}^{N} z_{i,k} y_i^4}{\sum_{i=1}^{N} z_{i,k}} - 3\left(\frac{\sum_{i=1}^{N} z_{i,k} y_i^2}{\sum_{i=1}^{N} z_{i,k}}\right)^2, \qquad (2)$$

where y_i is the image of $\mathbf{t}_i$ after projection.

The HC–KMC–SFNM–DCA projection takes four steps. (1) Apply HC on the data points that most likely belong to the cluster, i.e. the data points that have a bigger posterior probability of belonging to cluster k than to all other clusters. On the HC dendrogram, the user chooses a

distance threshold to cut the cluster into sub-clusters. Very small sub-clusters are merged into their nearest larger sub-clusters. (2) Run KMC on the data points using the sub-clusters obtained from HC as initialization. (3) Use the result of KMC to initialize an SFNM model in the visualization space, and run the EM algorithm to refine the model. The SFNM model and the corresponding EM algorithm take the form of Equation (12) and Equation (13), respectively. (4) DCA based on the weighted Fisher criterion uses the two eigenvectors associated with the largest eigenvalues of the weighted Fisher Scatter Matrix (wFSM) calculated based on the refined SFNM partition to project the cluster (Duda, *et al.*, 2001; Loog, *et al.*, 2001). Compared to the Fisher criterion, this weighted Fisher criterion confines the influence of class pairs that are well separated, and emphasizes on the class pairs that are overlapped. DCA based on the weighted Fisher criterion is expect to get a good total Bayesian classification accuracy, while a projection based on the Fisher criterion can be highly influenced by the well separated class pair, which is not so important for reducing the classification error. Suppose that K_k sub-clusters exist after the SFNM model fitting. Let $\boldsymbol{\mu}_{\mathbf{t},(k,j)}$ and $\boldsymbol{\Sigma}_{\mathbf{t},(k,j)}$ be the mean and covariance of sub-cluster j in the SFNM model. The wFSM is calculated by

$$\mathrm{wFSM} = \left(\mathbf{S}_{\mathbf{t},\mathrm{w}}\right)^{-1}\mathbf{S}_{\mathbf{t},\mathrm{b}}$$

$$\mathbf{S}_{\mathbf{t},\mathrm{w}} = \sum_{j=1}^{K_k} r_j \boldsymbol{\Sigma}_{\mathbf{t},(k,j)} \qquad \mathbf{S}_{\mathbf{t},\mathrm{b}} = \left(\sum_{i=1}^{K_k-1}\sum_{j=i+1}^{K_k} r_i r_j \omega\left(\Delta_{ij}\right)\left(\boldsymbol{\mu}_{\mathbf{t},(k,i)} - \boldsymbol{\mu}_{\mathbf{t},(k,j)}\right)\left(\boldsymbol{\mu}_{\mathbf{t},(k,i)} - \boldsymbol{\mu}_{\mathbf{t},(k,j)}\right)^T\right), \tag{3}$$

$$\omega\left(\Delta_{ij}\right) = \frac{1}{2\Delta_{ij}^2}\mathrm{erf}\left(\frac{\Delta_{ij}}{2\sqrt{2}}\right) \qquad \Delta_{ij} = \sqrt{\left(\boldsymbol{\mu}_{\mathbf{t},(k,i)} - \boldsymbol{\mu}_{\mathbf{t},(k,j)}\right)^T\left(\mathbf{S}_{\mathbf{t},\mathrm{w}}\right)^{-1}\left(\boldsymbol{\mu}_{\mathbf{t},(k,i)} - \boldsymbol{\mu}_{\mathbf{t},(k,j)}\right)}$$

where r_i is the sample proportion of sub-cluster i within cluster k, and erf(•) is the Gaussian error function. The two eigenvectors found in this way are not guaranteed to be orthogonal, so they need to be orthogonalized by the Gram–Schmidt process to achieve an affine projection.

LPP also works on the data points that most likely belong to the cluster. The projection directions are obtained through minimizing a compactness cost function, which is a weighted summation of the pair-wise distances between points in the projection space. The weights are assigned in a way that the distances between neighboring points have big weights while the distances between far apart points have small weights. Thus the minimization emphasizes on keeping the neighboring data points still close in the projection space and preserves the local data structure. The minimization is achieved by the generalized eigenvalue decomposition (He and Niyogi, 2004). The eigenvectors are also orthogonalized by the Gram–Schmidt process to form the projection matrix.

The APC–DCA projection follows the idea of using DCA to evaluate/confirm unsupervisedly obtained partitions, but the process is automatic. APC is applied on the data points that most likely belong to the cluster to find a partition. Being quite different from the KMC, which needs an initialization of cluster centers, APC simultaneously considers all data points as potential cluster centers. By viewing each data point as a node in a network, the affinity propagation method recursively transmits along edges of the networks real-valued messages, whose magnitude reflects the current affinity that one data point has for choosing another data point as its cluster center, until a good set of cluster centers and corresponding clusters emerges

(Frey and Dueck, 2007). The messages are updated to search for minima of the cost function, which is the sum of dissimilarities between data points and their cluster centers. It was shown that the affinity propagation method finds a "neighborhood minima" solution, i.e. the obtained solution is the best solution in a particular large region around it (Frey and Dueck, 2007; Weiss and Freeman, 2001). This condition is weaker than a global minimum but stronger than a local minimum. Compared to randomly initialized KMC, APC achieves a better cost function value in a time efficient manner (based on comparing a single run of APC with 10,000 runs of randomly initialized KMC) (Frey and Dueck, 2007). In addition, APC has an automatic model selection scheme. Here, DCA is also based on the wFSM calculated by Equation (3), but specialized for the "hard" case, because APC generates a hard partition, not a soft partition like SFNM fitting. Orthogonalization of the eigenvectors by the Gram–Schmidt process is needed to achieve an affine projection.

Obviously, VISDA's projection suite framework is scalable and extensive to incorporate various existing clustering and visualization algorithms to increase the chance of revealing data structure of interest. When all the projections are made and shown to the user, the user will be asked to select one projection that he/she thinks best reveals the data structure as the final visualization. An interesting question is how effective a particular projection is. It may be agreeable that the bench mark criteria in visual exploration are very different and difficult (Nielson, 1996). As shared by Bishop and Tipping (Bishop and Tipping, 1998), we believe that in data visualization there is no objective measure of quality, and so it is difficult to quantify the merit of a particular data visualization method, and the effectiveness of such a techniques is often highly data dependent and application dependent. A possible alternative is to perform a rigorous psychological evaluation using a simple and controlled environment, or to invite domain experts to directly evaluate the efficacy of the algorithm for a specified task. For the practical use of VISDA, the user has two guidelines for selecting the best projection. One guideline is human inspection/discernment on sub-cluster separability, i.e. if in a projection, the sub-clusters are well-separated and show clear data structure, this projection is a good projection. The other guideline is domain knowledge. If the user is certain about the relationship between some samples or genes under the particular experimental condition, he/she can choose a projection that favors this relationship. For example, in gene clustering, if several genes are well studied in previous researches and are known to be co-regulated and co-expressed under the particular experimental condition that produces the data, a projection that has these genes closely located is more preferred than a projection that has these genes located far apart.

Besides the two guidelines given above, from the experience of performing the experiments in this paper, authors also got some empirical understanding of using the projection suite. When the data size is pretty big, e.g. doing gene clustering of thousands of genes, the HC-KMC-SFNM-DCA, LPP, and APC-DCA projection may be slow and may even encounter the "out of memory" error, because they need to calculate square matrices with dimensionality of the data size in the clustering process. So for very big dataset, we did not use these three projections, but only use the PCA and PCA-PPM projections. When the data size is moderate, although all projections may be used as the final visualization, the authors used HC-KMC-SFNM-DCA and APC-DCA slightly more frequently, especially HC-KMC-SFNM-DCA. A possible reason is that

DCA finds the projection directions with which the obtained sub-clusters show best separability. Compared with APC-DCA, HC-KMC-SFNM-DCA can handle bigger dataset than APC-DCA and runs faster. It also includes more human data interaction and lets the user control the number of sub-clusters by cutting on the dendrogram of HC. When the data size is very small and the data dimensionality is high, i.e. the sample-to-dimensionality ratio is very low, the DCA based projections may always find a projection space that the sub-clusters show good separability, or even greatly squeeze the sub-cluster, so that it looks like one point in the projection space. In such cases, we normally did not choose DCA based projections.

2.1.2 Cluster decomposition in visualization space

We use the two-level hierarchical SFNM model to present the relationship between the lth and the l + 1th levels of the VISDA's hierarchical exploration. The probability density function for a two-level hierarchical SFNM model is formulated as:

$$\begin{gathered} \mathrm{f}\left(\mathbf{t}_i \middle| \boldsymbol{\theta}_{\mathbf{t}}, \boldsymbol{\pi}\right) = \sum_{k=1}^{K_l} \pi_k \sum_{j=1}^{K_{k,\,l+1}} \pi_{j|k}\, \mathrm{g}\left(\mathbf{t}_i \middle| \boldsymbol{\theta}_{\mathbf{t},(k,j)}\right) \\ \sum_{k=1}^{K_l} \pi_k = 1 \quad \text{and} \quad \sum_{j=1}^{K_{k,\,l+1}} \pi_{j|k} = 1 \end{gathered}, \tag{4}$$

where $K_{k,\ l+1}$ sub-clusters exist at level l + 1 for each cluster k of level l, π_k is the mixing proportion for cluster k in level l, $\pi_{j|k}$ is the mixing proportion for sub-cluster j within cluster k, g(•) is the Gaussian probability density function, and $\boldsymbol{\theta}_{\mathbf{t},\,(k,j)}$ are the associated parameters of sub-cluster j. When the cluster labels of the samples in level l are known, the conditional distribution is formulated as

$$\mathrm{f}\left(\mathbf{t}_i \middle| \boldsymbol{\theta}_{\mathbf{t}}, \boldsymbol{\pi}, \mathbf{r}_i\right) = \prod_{k=1}^{K_l} \left(\sum_{j=1}^{K_{k,\,l+1}} \pi_{j|k}\, \mathrm{g}\left(\mathbf{t}_i \middle| \boldsymbol{\theta}_{\mathbf{t},(k,j)}\right) \right)^{r_{i,k}}, \tag{5}$$

where $r_{i,\ k}$ is a binary cluster label indicator of sample i, i.e. one of $\{r_{i,\ 1}, \ldots, r_{i,K_l}\}$ is one and others are all zeros. In fact, we only have partial, probabilistic, information in the form of the posterior probability $z_{i,\ k}$ for cluster k having generated $\mathbf{t}_i$. Taking the expectation of the conditional log-likelihood and focusing only on cluster k, we get

$$\mathrm{L}(\mathbf{t} | \boldsymbol{\theta}_{\mathbf{t},k}, \boldsymbol{\pi}_k, \mathbf{z}_k) = \sum_{i=1}^{N} z_{i,k} \ln\left(\sum_{j=1}^{K_{k,\,l+1}} \pi_{j|k}\, \mathrm{g}\left(\mathbf{t}_i \middle| \boldsymbol{\theta}_{\mathbf{t},(k,j)}\right) \right). \tag{6}$$

According to the projection invariant property of normal distributions, i.e. a Gaussian distribution is still a Gaussian distribution after a linear projection, the projected data have an expectation of conditional log-likelihood given by

$$\mathrm{L}(\mathbf{x} | \boldsymbol{\theta}_{\mathbf{x},k}, \boldsymbol{\pi}_k, \mathbf{z}_k) = \sum_{i=1}^{N} z_{i,k} \ln\left(\sum_{j=1}^{K_{k,\,l+1}} \pi_{j|k}\, \mathrm{g}\left(\mathbf{x}_i \middle| \boldsymbol{\theta}_{\mathbf{x},(k,j)}\right) \right), \tag{7}$$

where $\mathbf{x} = \{\mathbf{x}_1, \mathbf{x}_2, \ldots, \mathbf{x}_N \mid \mathbf{x}_i \in \mathrm{R}^2, i = 1, 2, \ldots, N\}$ indicates all the projected data points, the subscript '**x**' indicates that these parameters model the data in the visualization space. Equation (7) is a weighted log-likelihood, whose value can be maximized or locally maximized by the

Expectation Maximization (EM) algorithm (Dempster, *et al.*, 1977). The EM algorithm iteratively performs the E-step and M-step to monotonically increase the log-likelihood until convergence. The E-step calculates the expectation of the samples' sub-cluster labels conditioned on the data and the current model parameters.

$$\text{E Step:}\quad z_{i,(k,j)} = z_{i,k}\frac{\pi_{j|k}\,\mathrm{g}(\mathbf{x}_i|\boldsymbol{\mu}_{\mathbf{x},(k,j)},\boldsymbol{\Sigma}_{\mathbf{x},(k,j)})}{\sum_{j=1}^{K_{k,l+1}}\pi_{j|k}\,\mathrm{g}(\mathbf{x}_i|\boldsymbol{\mu}_{\mathbf{x},(k,j)},\boldsymbol{\Sigma}_{\mathbf{x},(k,j)})}, \tag{8}$$

where $z_{i,(k,j)}$ is the posterior probability of data point $\mathbf{x}_i$ belonging to the jth sub-cluster in cluster k, $\boldsymbol{\mu}_{\mathbf{x},(k,j)}$ and $\boldsymbol{\Sigma}_{\mathbf{x},(k,j)}$ are the mean and covariance matrix of sub-cluster j in cluster k. The M step updates the model parameters using formulas given by

$$\text{M Step:}\quad \begin{array}{c}\pi_{j|k} = \sum_{i=1}^{N} z_{i,(k,j)} \Big/ \sum_{i=1}^{N} z_{i,k}, \qquad \boldsymbol{\mu}_{\mathbf{x},(k,j)} = \sum_{i=1}^{N} z_{i,(k,j)}\mathbf{x}_i \Big/ \sum_{i=1}^{N} z_{i,(k,j)} \\ \boldsymbol{\Sigma}_{\mathbf{x},(k,j)} = \sum_{i=1}^{N} z_{i,(k,j)}\left(\mathbf{x}_i-\boldsymbol{\mu}_{\mathbf{x},(k,j)}\right)\left(\mathbf{x}_i-\boldsymbol{\mu}_{\mathbf{x},(k,j)}\right)^T \Big/ \sum_{i=1}^{N} z_{i,(k,j)}\end{array}. \tag{9}$$

Models with different numbers of sub-clusters are initialized by the user and trained by the EM algorithm. The obtained partitions of all the models are displayed to the user as a reference for model selection. The MDL criterion is also utilized as a theoretical validation for model selection (Rissanen, 1978; Schwarz, 1978). Because the data size of the microarray gene expression dataset is usually small, the MDL model selection with Gaussian distributions has the tendency to select complex models in low dimensional space (Ridder, *et al.*, 2005). Based on our experimental experience and reference to (Liang, *et al.*, 1992; Ridder, *et al.*, 2005), we use a modified formula to calculate the description length given by

$$-\mathrm{L}(\mathbf{x}|\boldsymbol{\theta}_{\mathbf{x},k},\boldsymbol{\pi}_k,\mathbf{z}_k)+\frac{K_a\times N_k\times\ln(N_k)}{2(N_k-K_a)},\quad N_k=\sum_{i=1}^{N} z_{i,k} \text{ and } K_a = 6K_{k,l+1}-1, \tag{10}$$

where K_a and N_k are the number of free adjustable parameters and the effective number of data points in the cluster, respectively. This modified MDL formula not only eases the trend to overestimate the sub-cluster number when the data size is small, but also is asymptotically consistent with the classical MDL formula, which is

$$-\mathrm{L}(\mathbf{x}|\boldsymbol{\theta}_{\mathbf{x},k},\boldsymbol{\pi}_k,\mathbf{z}_k)+\frac{K_a}{2}\ln(N_k). \tag{11}$$

2.1.3 The full dimensional model and its training algorithm

The probability density function of the SFNM model is

$$\mathrm{f}\left(\mathbf{t}_i|\boldsymbol{\theta}_{\mathbf{t}},\boldsymbol{\pi}\right)=\sum_{k=1}^{K_{l+1}}\pi_k\,\mathrm{g}\left(\mathbf{t}_i|\boldsymbol{\theta}_{\mathbf{t},k}\right),\quad\text{and}\quad\sum_{k=1}^{K_{l+1}}\pi_k=1, \tag{12}$$

where K_{l+1} is the number of clusters at level l+1 and equal to $\sum_{k=1}^{K_l} K_{k,l+1}$, $\boldsymbol{\theta}_{\mathbf{t},k}$ is the set of parameters of the kth cluster at level l+1. The EM algorithm to refine the model is

$$\text{E-Step:} \quad z_{i,k} = \frac{\pi_k \, \mathrm{g}(\mathbf{t}_i | \boldsymbol{\mu}_{\mathbf{t},k}, \boldsymbol{\Sigma}_{\mathbf{t},k})}{\sum_{k=1}^{K_{l+1}} \pi_k \, \mathrm{g}(\mathbf{t}_i | \boldsymbol{\mu}_{\mathbf{t},k}, \boldsymbol{\Sigma}_{\mathbf{t},k})}$$

$$\text{M-Step:} \quad \pi_k = \sum_{i=1}^{N} z_{i,k} \Big/ N, \qquad \boldsymbol{\mu}_{\mathbf{t},k} = \sum_{i=1}^{N} z_{i,k} \mathbf{t}_i \Big/ \sum_{i=1}^{N} z_{i,k}, \qquad \boldsymbol{\Sigma}_{\mathbf{t},k} = \sum_{i=1}^{N} z_{i,k} \left(\mathbf{t}_i - \boldsymbol{\mu}_{\mathbf{t},k}\right)\left(\mathbf{t}_i - \boldsymbol{\mu}_{\mathbf{t},k}\right)^T \Big/ \sum_{i=1}^{N} z_{i,k} \quad (13)$$

The E-step and the M-step run iteratively until convergence.

2.2 Algorithm extension for sample clustering

Let $\{g_1, \ldots, g_N\}$ be the expression values of a gene. The variance of $\{g_1, \ldots, g_N\}$ is calculated by

$$\frac{1}{N-1}\sum_{i=1}^{N}\left(g_i - \frac{1}{N}\sum_{j=1}^{N} g_j\right)^2 . \quad (14)$$

The absolute difference between the minimum and maximum gene expression values across all the samples is calculated by

$$\max\{g_1, \ \cdots, \ g_N\} - \min\{g_1, \ \cdots, \ g_N\}. \quad (15)$$

These two criteria can be used to identify and then remove constantly expressed genes. A rank of all the genes for each of the two variation criteria can be formed.

Discrimination power analysis uses a 1-D SFNM model to fit the gene's expression values. The probability density function of the model and its corresponding EM algorithm take the form of Equation (12) and Equation (13), respectively, but in 1-D space. To determine the model order, we followed the iterative procedure in (Miller and Browning, 2003). The SFNM model is initialized with a high model order (much bigger than the true component number), with randomly chosen means and uniform variances. In each iteration, we (one-by-one) trial-delete each component and rerun the fitting algorithm. The component whose removal yields minimum description length will be permanently removed. This iterative process ends when only one component remains, and the optimum model order is then determined by comparing the description length (or modified description length if the sample size is small) values of solutions in the sequence. Once the SFNM model is trained, genes with single component mixture are removed, because they do not support any cluster structure. For the other genes, the accuracy in classifying samples to components resulting from applying the Maximum A Posteriori probability (MAP) rule (based on the samples' posterior probabilities for belonging to the components) quantifies the gene's discrimination power. Thus, a rank of genes according to their discrimination power can be constructed.

The classification accuracy of a K-component mixture has a low limit of $1/K$, which decreases when K increases. However, this may not impact but actually may improve the clustering performance of VISDA due to the following reasons. (1) VISDA performs a hierarchical exploration process where the data decomposition models are initialized by data

structures presented in multiple subspaces. The combinatory power of multiple subspaces helps VISDA to detect all clusters. Thus a single gene or a single subspace is not required to discern among many or all the clusters. (2) With a fewer number of components, the statistics of each component can be better estimated, which is important for sample clustering on genomic data, where the sample size is normally small. (3) With a fewer number of components in the subspace, the local decomposition problem at each node of the VISDA hierarchical exploration process is simpler to solve. (4) In many genomic research applications where gene expression values are required to be quantized or genes are analyzed in terms of states, genes are normally assumed to take very few (two or three) discrete values or states, which corresponds to down-regulation and up-regulation or low, middle, and high states (Xing and Karp, 2001).

Besides the two kinds of non-informative genes discussed above, “redundant” genes (genes that are highly correlated with other genes) provide only limited additional separability between sample clusters. However, this limited additional separability may in fact greatly improve the achievable partition accuracy (Guyon and Elisseeff, 2003). Thus, we take removal of redundant genes as an optional step. If the dimensionality of the gene space after variation filtering and discrimination power filtering can not be well handled by the clustering algorithm (i.e. if the samples-to-genes ratio is not sufficiently large), we suggest removing highly correlated genes. Here, we provide a simple scheme to remove redundant genes. In the gene list resulting from variation filtering and discrimination power analysis, keep the most discriminative gene and remove the genes that are highly correlated with it, then keep the second most discriminative gene in the remaining list and remove the genes that are highly correlated with this gene. Keep performing this procedure until no further removal can be done. The correlation between genes can be measured by the Pearson correlation coefficient or mutual information normalized by entropy. A threshold needs to be set to identify the highly correlated genes.

2.3 Algorithm extension for phenotype clustering

Suppose that the exploration process has proceeded to the lth level and level l has K_l phenotype clusters. For each phenotype cluster k (k = 1, …, K_l) at level l, we do the following to visualize and decompose the cluster.

2.3.1 Locally discriminative gene subspace and visualization

Let $\mathbf{t} = \{\mathbf{t}^{(1)}, \ldots, \mathbf{t}^{(Q_k)}\}$ denote the phenotypes in the cluster. Q_k is the number of phenotypes in cluster k. $\mathbf{t}^{(q)} = \{\mathbf{t}_i^{(q)}, i = 1, 2, \ldots, N^{(q)}\}$ (q = 1, …, Q_k) is the set of samples in phenotype q. To achieve an effective visualization, we first use a supervised gene selection method to select top discriminative genes to form a locally discriminative gene subspace, and then project the samples from the discriminative gene subspace to a 2-D visualization space through DCA.

Let μ_q and σ_q be the mean and standard deviation of a gene’s expression values in phenotype q. A gene’s discrimination power is measured by

$$\left(\sum_{q=1}^{Q_k-1}\sum_{m=q+1}^{Q_k} r_q r_m \left(\mu_q-\mu_m\right)^2\right)\Big/\left(\sum_{q=1}^{Q_k} r_q\sigma_q^2\right), \tag{16}$$

where r_q is the sample proportion of phenotype q and equal to $N^{(q)}\Big/\sum_{q=1}^{Q_k}N^{(q)}$. According to the discrimination power, top discriminative genes can be selected to form a locally discriminative gene subspace. The number of selected genes is n_gQ_k, where n_g is the number of selected genes per phenotype. We use DCA based on the Fisher criterion to find a projection matrix to project the samples from the gene subspace onto a 2-D visualization space. The projection matrix is obtained by orthogonalizing the eigenvectors associated with the largest two eigenvalues of the Fisher Scatter Matrix (FSM) that is calculated based on the phenotypic categories (Duda, *et al.*, 2001). Here, we use the Fisher criterion not the weighted Fisher criterion (i.e. do not weight the phenotype pairs in the objective function) for the purpose of preserving original data structure by treating all phenotype pairs fair. Thus the identified TOP will not be distorted. Let $\boldsymbol{\mu}_{\mathbf{s},i}$ and $\boldsymbol{\Sigma}_{\mathbf{s},i}$ be the mean and covariance matrix of phenotype i in the gene subspace. The FSM is calculated by

$$\begin{aligned}&\mathrm{FSM}=\left(\mathbf{S}_{\mathbf{s},\mathrm{w}}\right)^{-1}\mathbf{S}_{\mathbf{s},\mathrm{b}}\\&\mathbf{S}_{\mathbf{s},\mathrm{w}}=\sum_{q=1}^{Q_k} r_q\boldsymbol{\Sigma}_{\mathbf{s},q}\qquad \mathbf{S}_{\mathbf{s},\mathrm{b}}=\left(\sum_{q=1}^{Q_k-1}\sum_{m=q+1}^{Q_k} r_q r_m\left(\boldsymbol{\mu}_{\mathbf{s},q}-\boldsymbol{\mu}_{\mathbf{s},m}\right)\left(\boldsymbol{\mu}_{\mathbf{s},q}-\boldsymbol{\mu}_{\mathbf{s},m}\right)^T\right)\end{aligned}. \tag{17}$$

2.3.2 Cluster decomposition in the visualization space

In the visualization space, we use a class-pooled finite normal mixtures model to fit and decompose the cluster. Let $\mathbf{x}$ = {$\mathbf{x}^{(1)}$, …, $\mathbf{x}^{(q)}$, …, $\mathbf{x}^{(Q_k)}$} denote the projected samples from each phenotype. $\mathbf{x}^{(q)}$ = {$\mathbf{x}_i^{(q)}$, i = 1, 2, …, $N^{(q)}$} is the set of samples in phenotype q. The probability density function for all samples from phenotype q is

$$\mathrm{f}\left(\mathbf{x}^{(q)}\middle|\boldsymbol{\theta}_{\mathbf{x}},\boldsymbol{\pi}\right)=\sum_{j=1}^{K_{k,l+1}}\pi_j\prod_{i=1}^{N^{(q)}}\mathrm{g}\left(\mathbf{x}_i^{(q)}\middle|\boldsymbol{\theta}_{\mathbf{x},j}\right)\quad\text{and}\quad\sum_{j=1}^{K_{k,l+1}}\pi_j=1, \tag{18}$$

where cluster k at level l is decomposed into $K_{k,\,l+1}$ sub-clusters at level l + 1, π_j and $\boldsymbol{\theta}_{\mathbf{x},j}$ are the mixing proportion and parameters associated with sub-cluster j. The EM algorithm used to train this model is formulated as

$$\begin{aligned}&\text{E Step:}\quad z_{(q),j}=\pi_j\prod_{i=1}^{N^{(q)}}\mathrm{g}\left(\mathbf{x}_i^{(q)}\middle|\boldsymbol{\theta}_{\mathbf{x},j}\right)\Big/\sum_{j=1}^{K_{k,l+1}}\pi_j\prod_{i=1}^{N^{(q)}}\mathrm{g}\left(\mathbf{x}_i^{(q)}\middle|\boldsymbol{\theta}_{\mathbf{x},j}\right)\\&\text{M Step:}\quad \pi_j=\frac{1}{Q_k}\sum_{q=1}^{Q_k}z_{(q),j}\qquad \boldsymbol{\mu}_{\mathbf{x},j}=\sum_{q=1}^{Q_k}\left(z_{(q),j}\sum_{i=1}^{N^{(q)}}\mathbf{x}_i^{(q)}\right)\Big/\sum_{q=1}^{Q_k}z_{(q),j}N^{(q)},\\&\qquad\qquad \boldsymbol{\Sigma}_{\mathbf{x},j}=\sum_{q=1}^{Q_k}z_{(q),j}\sum_{i=1}^{N^{(q)}}\left(\mathbf{x}_i^{(q)}-\boldsymbol{\mu}_{\mathbf{x},j}\right)\left(\mathbf{x}_i^{(q)}-\boldsymbol{\mu}_{\mathbf{x},j}\right)^T\Big/\sum_{q=1}^{Q_k}z_{(q),j}N^{(q)}\end{aligned} \tag{19}$$

where $z_{(q),j}$ is the posterior probability of phenotype q (all samples from phenotype q) belonging to sub-cluster j, and $\boldsymbol{\mu}_{\mathbf{x},j}$ and $\boldsymbol{\Sigma}_{\mathbf{x},j}$ are the mean and covariance matrix of sub-cluster j in the

visualization space. To select the “optimal” model from models with different number of sub-clusters, the MDL model selection criterion is also applied for theoretical validation. For the small sample size case, the description length is calculated by

$$-\mathrm{L}(\mathbf{x}|\boldsymbol{\theta}_{\mathbf{x}},\boldsymbol{\pi})+\frac{K_a\times N\times\ln(N)}{2(N-K_a)}, \tag{20}$$

where $\mathrm{L}(\mathbf{x}|\boldsymbol{\theta}_{\mathbf{x}}, \boldsymbol{\pi})$ is the log-likelihood, K_a is the number of free adjustable parameters, and N is the number of samples. $\mathrm{L}(\mathbf{x}|\boldsymbol{\theta}_{\mathbf{x}}, \boldsymbol{\pi})$, K_a, and N are given by

$$\begin{gathered}\mathrm{L}(\mathbf{x}|\boldsymbol{\theta}_{\mathbf{x}},\boldsymbol{\pi})=\sum_{q=1}^{Q_k}\ln\sum_{j=1}^{K_{k,l+1}}\pi_j\prod_{i=1}^{N^{(q)}}\mathrm{g}\left(\mathbf{x}_i^{(q)}\middle|\boldsymbol{\theta}_{\mathbf{x},j}\right)\\ N=\sum_{q=1}^{Q_k}N^{(q)}\quad\text{and}\quad K_a=6K_{k,l+1}-1\end{gathered} \tag{21}$$

The user can override the MDL model selection by specifying the number of sub-clusters according to his/her justification.

3. Additional Discussion

In further research, some modifications can be made to improve VISDA. For example, 3-D visualization and model initialization can be implemented without theoretical obstacles. Nonlinear projections may also be used to visualize data cluster, but two things need to be noted. (1) Suitable data models need to be defined in the visualization space and in the original data space. (2) A corresponding inverse (or approximate inverse) transform of model parameters from the visualization space to the original data space must also be defined. For phenotype clustering, in the current version of VISDA, the gene subspace is formed by selecting individually discriminative genes. Because the jointly most discriminative gene set does not necessarily solely consist of the genes that are most discriminative individually (Jain, *et al.*, 2000), an improvement based on selecting jointly discriminative genes can be made using methods such as (Xuan, *et al.*, 2007), at the cost of increased computation time.

4. SOFTWARE AVAILABILITY

VISDA is a toolkit of caBIG™ (https://cabig.nci.nih.gov/). Open source caBIG™ VISDA software package is free available at https://gforge.nci.nih.gov/projects/visda. (Wang, *et al.*, 2007) is an application note introducing the caBIG™ VISDA software. An updated version of the installation guide for this open source software is available at http://www.cbil.ece.vt.edu/software/VISDA_InstallationGuide.pdf. Matlab code implementing a full functional version of VISDA is free available at http://www.cbil.ece.vt.edu/software/VISDA_Package.zip.

References

Ben-Dor, A.*, et al.* (1999) Clustering Gene Expression Patterns, *J. Comput. Biol.*, **6**, 281-297.
Benito, M.*, et al.* (2004) Adjustment of systematic microarray data biases, *Bioinformatics*, **20**, 105-114.
Bhattacharjee, A.*, et al.* (2001) Classification of human lung carcinomas by mRNA expression profiling reveals distinct adenocarcinoma subclasses, *Proc. Natl. Acad. Sci. U.S.A.*, **98**, 13790-13795.
Bishop, C.M. (1995) *Neural Networks for Pattern Recognition*. Clarendon Press, Oxford University.
Bishop, C.M. and Tipping, M.E. (1998) A hierarchical latent variable model for data visualization, *IEEE Trans. Pattern Anal. Mach. Intell.*, **20**, 282-293.
Bloom, G.*, et al.* (2004) Multi-platform, multi-site, microarray-based human tumor classification, *Am. J. Pathol.*, **164**, 9-16.
Brown, M.P.S.*, et al.* (2000) Knowledge-based analysis of microarray gene expression data using support vector machines, *Proc. Natl. Acad. Sci. USA*, **97**, 262-267.
Chien, Y. (1978) *Interactive Pattern Recognition* Marcel Dekker.
Clarke, R.*, et al.* (2008) The properties of high-dimensional data spaces: implications for exploring gene and protein expression data, *Nat. Rev. Cancer*, **8**, 37-49.
Dempster, A.P.*, et al.* (1977) Maximum likelihood from incomplete data via the EM algorithm., *J. R. Statist. Soc., Series B*, **34**, 1-38.
Duda, R.O.*, et al.* (2001) *Pattern Classification*. John Wiley & Sons Inc.
Dy, J. and Brodley, C. (2000) Feature subset selection and order identification for unsupervised learning, *Proc. 17th Intl. Conf. Mach. Learn.*, 247-254.
Frey, B.J. and Dueck, D. (2007) Clustering by passing messages between data points, *Science*, **315**, 972-976.
Giordano, T.J.*, et al.* (2001) Organ-specific molecular classification of primary lung, colon, and ovarian adenocarcinomas using gene expression profiles, *Am. J. Pathol.*, **159**, 1231-1238.
Graham, M.W. and Miller, D.J. (2006) Unsupervised learning of parsimonious mixtures on large spaces with integrated feature and component selection, *IEEE Trans. Signal Proces.*, **54**, 1289-1303.
Guyon, I. and Elisseeff, A. (2003) An introduction to variable and feature selection, *J. Mach. Learn. Res.*, **3**, 1157-1182.
Hartigan, J. (1975) *Clustering algorithms*. Wiley Publishers, New York.
He, X. and Niyogi, P. (2004) Locality preserving projections. In Thrun, S.*, et al.* (eds), *Advances in Neural Information Processing Systems 16*. MIT Press, Cambridge, M.A.
Hyvärinen, A.*, et al.* (2001) *Independent Component Analysis* Wiley-Interscience.
Jain, A.K.*, et al.* (2000) Statistical pattern recognition: a review, *IEEE Trans. Pattern Anal. Mach. Intell.*, **22**, 4-38.
Jiang, D.*, et al.* (2004) Cluster analysis for gene expression data: a survey, *IEEE Trans. Know. Data Eng.*, **16**, 1370-1386.
Khan, J.*, et al.* (2001) Classification and diagnostic prediction of cancers using gene expression profiling and artificial neural networks, *Nat. Med.*, **7**, 673-679.

Lange, T., *et al.* (2004) Stability-based validation of clustering solutions, *Neural Comput.*, **16**, 1299-1323
Liang, Z., *et al.* (1992) Parameter estimation of finite mixtures using the EM algorithm and information criteria with application to medical image processing, *IEEE Trans. Nucl. Sci.*, **39**, 1126-1133.
Loog, M., *et al.* (2001) Multiclass linear dimension reduction by weighted pairwise fisher criteria, *IEEE Trans. Pattern Anal. Mach. Intell.*, **23**, 762-766.
Miller, D.J. and Browning, J. (2003) A mixture model and EM-based algorithm for class discovery, robust classification, and outlier rejection in mixed labeled/unlabeled data sets, *IEEE Trans. Pattern Anal. Mach. Intell.*, **25**, 1468-1483.
Miller, D.J., *et al.* (2008) Emergent unsupervised clustering paradigms with potential application to bioinformatics, *Front. Biosci.*, **13**, 677-690.
Nielson, G.M. (1996) Chanllenges in visualization research, *IEEE Trans. Vis. Comput. Graph.*, **2**, 97-99.
Pan, W. (2006) Incorporating gene functions as priors in model-based clustering of microarray gene expression data, *Bioinformatics*, **22**, 795-801.
Qu, Y. and Xu, S. (2004) Supervised cluster analysis for microarray data based on multivariate Gaussian mixture, *Bioinformatics*, **20**, 1905-1913.
Ridder, F.D., *et al.* (2005) Modified AIC and MDL model selection criteria for short data records, *IEEE Trans. Instrum. Meas.*, **54**, 144-150.
Rissanen, J. (1978) Modeling by shortest data description, *Automatica*, **14**, 465-471.
Rose, K. (1998) Deterministic annealing for clustering, compression,classification, regression, and related optimization problems, *Proc. IEEE*, **86**, 2210-2239.
Roth, V. and Lange, T. (2004) Bayesian class discovery in microarray datasets, *IEEE Trans. Biomed. Eng.*, **51**, 707-718.
Schwartz, D.R., *et al.* (2002) Gene expression in ovarian cancer reflects both morphology and biological behavior, distinguishing clear cell from other poor-prognosis ovarian carcinomas, *Cancer Res.*, **62**, 4722-4729.
Schwarz, G. (1978) Estimating the dimension of a model, *Ann. Statistics*, **6**, 461-464.
Shedden, K.A., *et al.* (2003) Accurate molecular classification of human cancers based on gene expression using a simple classifier with a pathological tree-based framework, *Am. J. Pathol.*, **163**, 1985-1995.
Strehl, A. and Ghosh, J. (2002) Cluster ensembles -- a knowledge reuse framework for combining partitionings, *J. Mach. Learn. Res.*, **3**, 583-617.
Su, A.I., *et al.* (2001) Molecular classification of human carcinomas by use of gene expression signatures, *Cancer Res.*, **61**, 7388-7393.
Tipping, M. and Bishop, C. (1999) Mixtures of probabilistic principal component analyzers, *Neural Comput.*, **11**, 443-482.
Topchy, A., *et al.* (2005) Clustering ensembles: models of consensus and weak partitions, *IEEE Trans. Pattern Anal. Mach. Intell.*, **27**, 1866-1881.
Wang, J., *et al.* (2007) VISDA: an open-source caBIG analytical tool for data clustering and beyond, *Bioinformatics*, **23**, 2024-2027.
Wang, Y., *et al.* (2008) Approaches to working in high dimensional data spaces: gene expression microarray, *Brit. J. Cancer*, **98**, 1023-1028.

Wang, Z., *et al.* (2003) Discriminatory mining of gene expression microarray data, *J. VLSI Signal Processing* **35**, 255-272.

Weiss, Y. and Freeman, W.T. (2001) On the optimality of solutions of the max-product belief-propagation algorithm in arbitrary graphs, *IEEE Trans. Inform. Theory*, **47**, 736-744.

Xing, E.P. and Karp, R.M. (2001) CLIFF: clustering of high-dimensional microarray data via iterative feature filtering using normalized cuts, *Bioinformatics*, **17**, 306-315.

Xu, R. and Wunsch, D. (2005) Survey of clustering algorithms, *IEEE Trans. Neural Networks*, **16**, 645-678.

Xuan, J., *et al.* (2007) Gene selection for multiclass prediction by weighted fisher criterion, *EURASIP J. Bioinform. and Syst. Biol.*, **2007**.

Zhu, X. (2006) Semi-supervised learning literature survey. *Computer Science Technical Report 1530*. U. of Wisconsin.

Zhu, Y., *et al.* (2006) Phenotypic-specific gene module discovery using a diagnostic tree and caBIG™ VISDA. *28th IEEE EMBS Annual Int. Conf.*, New York City.

Zhu, Y., *et al.* (2008) A ground truth based comparative study on clustering of gene expression data, *Front. Biosci.*, **13**, 3839-3849.

Zou, J. and Nagy, G. (2006) Human-computer interaction for complex pattern recognition problems. In Basu, M. and Ho, T.K. (eds), *Data Complexity in Pattern Recognition*. Springer, 271-286.

Supplement Table 1. A summary of the datasets used in the evaluation experiment.

Diagnostic task	Biological category (number of samples in the category)	Class number / number of selected genes	Source
Synthetic data	4 Gaussian distributed classes, each class has 100 samples	4 / 3	
Small round blue cell tumours	Ewing sarcoma (29), burkitt lymphoma (11), neuroblastoma (18), rhabdomyosarcoma (25)	4 / 60	(Khan, et al., 2001)
Multiple human tumour types	Prostate cancer (10), breast cancer (12), kidney cancer (10), lung cancer (17)	4 / 7	(Su, et al., 2001)
Lung cancer sub-types and normal tissues	Adenocarcinomas (16), normal lung (17), squamous cell lung carcinomas (21), pulmonary carcinoids (20)	4 / 13	(Ben-Dor, et al., 1999; Bhattacharjee, et al., 2001)
Classification of multiple human cancer types	Brain cancer (73), colon cancer (60), lung cancer (91), ovary cancer (119, including 6 uterine cancer samples)	4 / 8	(Giordano, et al., 2001)
Ovarian cancer sub-types and clear cell	Ovarian serous (29), ovarian mucinous (10), ovarian endometrioid (36), clear ovarian cell (9)	4 / 25	(Schwartz, et al., 2002; Shedden, et al., 2003)
Human cancer data from multi-platforms and multi-sites	Breast cancer (22), central-nervous meduloblastoma (57), lung-squamous cell carcinoma (20), prostate cancer (39)	4 / 15	(Bloom, et al., 2004)
Human cancer data from multi-platforms and multi-sites	Central-nervous glioma (10), lung cancer (58), lung-squamous cell carcinoma (21) lymphoma-large B cell (11), prostate cancer (41)	5 / 20	(Bloom, et al., 2004)

Supplement Table 2. A brief introduction of the muscle dystrophy dataset.

Phenotype	Sample Number	Description
JDM	25	Juvenile dermatomyositis
FKRP	7	Fukutin related protein deficiency
DMD	10	Duchenne muscular dystrophy, dystrophin deficiency
BMD	5	Becker muscular dystrophy, hypomorphic for dystrophin
Dysferlin	10	Dysferlin deficiency, putative vesicle traffic defect
Calpain III	10	Calpain III deficiency
FSHD	14	Fascioscapulohumeral dystrophy
AQM	5	Acute quadriplegic myopathy
HSP	4	Spastin haploinsufficiency, microtubule traffic defect
Lamin A/C	4	Emery dreifuss muscular dystrophy, missense mutations
Emerin	4	Emery dreifuss muscular dystrophy, emerin deficient
ALS	9	Amyotrophic lateral sclerosis
NHM	18	Normal skeletal muscle

Supplement Table 3. A brief introduction of the multi-class cancer dataset.

Phenotype	Sample Number
Breast Cancer	11
Prostate Cancer	10
Lung Cancer	11
Colon Cancer	11
Lymphoma Cancer	22
Melanoma Cancer	10
Bladder Cancer	11
Uterus Cancer	10
Leukemia Cancer	30
Kidney Cancer	11
Pancreas Cancer	11
Ovary Cancer	11
Mesothelioma Cancer	11
Central Nervous System Cancer	20
